# Base excision repair of the *N*-(2-deoxy-d-*erythro*-pentofuranosyl)-urea lesion by the hNEIL1 glycosylase

**DOI:** 10.1093/nar/gkad164

**Published:** 2023-04-04

**Authors:** Rachana Tomar, Irina G Minko, Pankaj Sharma, Andrew H Kellum, Li Lei, Joel M Harp, T M Iverson, R Stephen Lloyd, Martin Egli, Michael P Stone

**Affiliations:** Department of Chemistry and the Vanderbilt-Ingram Cancer Center, Vanderbilt University, Station B Box 351822, Nashville, TN 37235, USA; Oregon Institute of Occupational Health Sciences, Oregon Health & Science University, 3181 SW Sam Jackson Park Rd., Portland, OR 97239, USA; Department of Pharmacology, Vanderbilt University, Nashville, TN 37232, USA; Department of Chemistry and the Vanderbilt-Ingram Cancer Center, Vanderbilt University, Station B Box 351822, Nashville, TN 37235, USA; Department of Biochemistry, School of Medicine, and the Vanderbilt-Ingram Cancer Center, Vanderbilt University, Nashville, TN 37232, USA; Department of Biochemistry, School of Medicine, and the Vanderbilt-Ingram Cancer Center, Vanderbilt University, Nashville, TN 37232, USA; Department of Pharmacology, Vanderbilt University, Nashville, TN 37232, USA; Department of Biochemistry, School of Medicine, and the Vanderbilt-Ingram Cancer Center, Vanderbilt University, Nashville, TN 37232, USA; Oregon Institute of Occupational Health Sciences, Oregon Health & Science University, 3181 SW Sam Jackson Park Rd., Portland, OR 97239, USA; Department of Molecular and Medical Genetics, Oregon Health & Science University, 3181 SW Sam Jackson Park Rd., Portland, OR 97239, USA; Department of Biochemistry, School of Medicine, and the Vanderbilt-Ingram Cancer Center, Vanderbilt University, Nashville, TN 37232, USA; Department of Chemistry and the Vanderbilt-Ingram Cancer Center, Vanderbilt University, Station B Box 351822, Nashville, TN 37235, USA

## Abstract

The *N*-(2-deoxy-d-*erythro*-pentofuranosyl)-urea DNA lesion forms following hydrolytic fragmentation of *cis*-5*R*,6*S*- and *trans*-5*R*,6*R*-dihydroxy-5,6-dihydrothymidine (thymine glycol, Tg) or from oxidation of 7,8-dihydro-8-oxo-deoxyguanosine (8-oxodG) and subsequent hydrolysis. It interconverts between α and β deoxyribose anomers. Synthetic oligodeoxynucleotides containing this adduct are efficiently incised by unedited (K242) and edited (R242) forms of the hNEIL1 glycosylase. The structure of a complex between the active site unedited mutant CΔ100 P2G hNEIL1 (K242) glycosylase and double-stranded (ds) DNA containing a urea lesion reveals a pre-cleavage intermediate, in which the Gly2 N-terminal amine forms a conjugate with the deoxyribose C1′ of the lesion, with the urea moiety remaining intact. This structure supports a proposed catalytic mechanism in which Glu3-mediated protonation of O4′ facilitates attack at deoxyribose C1′. The deoxyribose is in the ring-opened configuration with the O4′ oxygen protonated. The electron density of Lys242 suggests the ‘residue 242-in conformation’ associated with catalysis. This complex likely arises because the proton transfer steps involving Glu6 and Lys242 are hindered due to Glu6-mediated H-bonding with the Gly2 and the urea lesion. Consistent with crystallographic data, biochemical analyses show that the CΔ100 P2G hNEIL1 (K242) glycosylase exhibits a residual activity against urea-containing dsDNA.

## Graphical Abstract

**Graphical Abstract:**
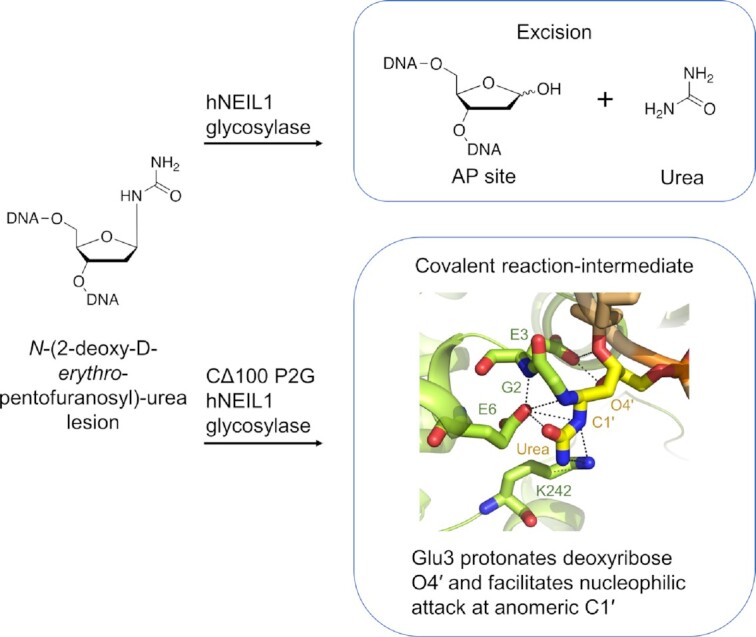
Base excision repair of a urea lesion by the hNEIL1 glycosylase. Crystallography of CΔ100 P2G mutant hNEIL1 (K242) and damaged DNA reveals the Gly2 N-terminal amine conjugated to deoxyribose C1′, showing Glu3-mediated protonation at O4′.

## INTRODUCTION

DNA is susceptible to structural modifications following oxidative damage (1–4). These arise both from exogenous and endogenous oxidants (5–7). Such damage is primarily repaired by base excision repair (BER) (8–11), with DNA glycosylases initiating the pathway (12–18). The mechanistic enzymology of BER and the recognition of DNA damage by glycosylases is therefore of continuing interest. Human NEIL1 glycosylase (hNEIL1), a bifunctional glycosylase that belongs to the Fpg/Nei family, initiates BER of a variety of DNA lesions ranging from the diastereomeric *cis*-5*R*,6*S*- and *trans*-5*R*,6*R*-dihydroxy-5,6-dihydrothymidine (thymine glycol, Tg) lesions, common oxidation damage products (19–21), to bulkier AFB_1_-Fapy dG lesions (22–46). Editing of pre-mRNA encoding h*NEIL1* results in an amino acid change at position 242, from lysine in the unedited form of the glycosylase to arginine in the edited form (34,37,44). This change has been shown to modulate the rates of base excision on a range of substrates (34,37,43,44,46,47), with the unedited form being significantly more efficient in excising Tg lesions than the edited form (34,43,46,47). hNEIL1 also possesses apurinic/apyrimidinic (AP) lyase activity that catalyzes β-δ elimination (48), generating a single-nucleotide gap (8,22–24,31), with both 5′ and 3′ phosphates (8,12,16). The ability of hNEIL1 to initiate repair on substrates in which the lesions are located in bubble or forked DNA structures (25,35,39,44) suggests that hNEIL1 plays a role in removing oxidative damage during replication, as part of a BER multi-protein complex (13,49–51). This idea was reinforced by observations of protein-protein interactions between hNEIL1 and various replication-associated proteins including PCNA, FEN1 and RPA (52–55).

The hNEIL1 glycosylase has been the target of several structural investigations (37,47,56–58) following the determination of the initial crystal structure of hNEIL1 (apo enzyme) (59). Using active-site mutants having a Pro to Gly replacement at the second residue of C-terminally truncated versions of both edited and unedited hNEIL1 (P2G), Zhu *et al.* (47) analyzed co-crystals with DNA containing Tg or the stable AP site analog tetrahydrofuran (THF). The resulting structures led them (47,58) to propose a catalytic mechanism by which hNEIL1 excises Tg lesions. It was proposed that specific substrate recognition loop residues in hNEIL1, particularly Lys242 in the unedited glycosylase or Arg242 in the edited glycosylase, play critical roles in recognizing oxidative DNA damage by exploiting lactam-lactim tautomerism in a range of substrates (47,58). For Tg, a lower free energy ‘242-in conformation’, referring to the positioning of either Lys242 or Arg242 within the active site complex, seemed essential for substrate recognition and discrimination.

**Scheme 1.**
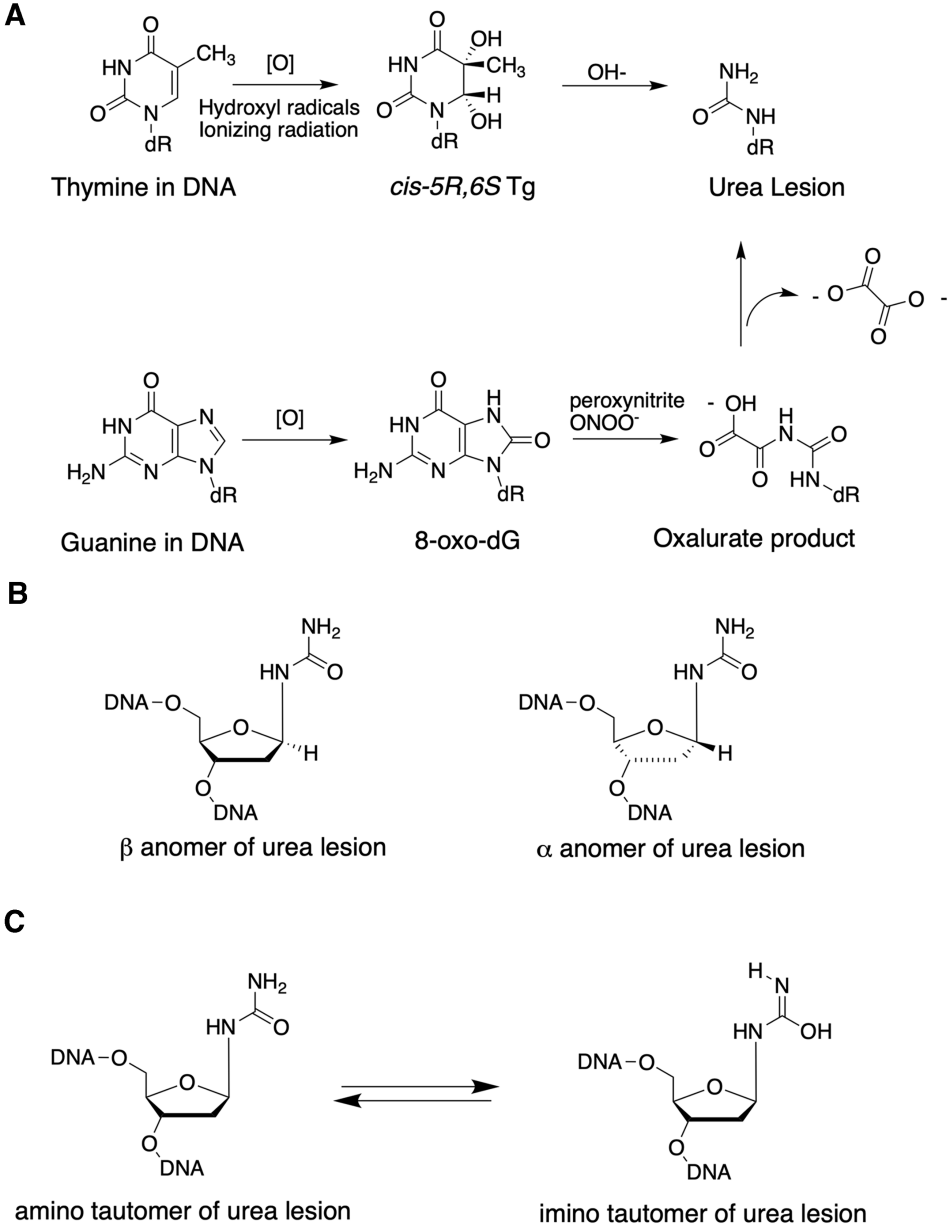
(**A**) The urea lesion is a secondary oxidative DNA damage product, arising from Tg or 8-oxo-dG. (**B**) α and β anomers of the urea lesion. (**C**) Amino and imino tautomers of the urea lesion.

The commonly formed diastereomeric Tg lesions may undergo hydrolytic fragmentation to form the urea lesion, *N*-(2-deoxy-D-*erythro*-pentofuranosyl)-urea (Scheme 1A) (60–64). The latter may also form following oxidation of 7,8-dihydro-8-oxo-deoxyguanosine (8-oxo-dG) and subsequent hydrolysis (Scheme 1A) (65). The potential for epimerization between α and β anomers of the urea lesion (Scheme 1B) has been reported at the nucleoside level (66,67). Like Tg, urea lesions have the potential to tautomerize (Scheme 1C). The urea lesion has also been reported to be a substrate for hNEIL1 (27). We envisioned that structural analyses of a complex between the urea lesion and hNEIL1 might not only provide insight into the mechanism by which this glycosylase excises urea lesions, but also advance an understanding of the excision of more abundant Tg and other lesions. An evaluation of the relative activities of edited vs. unedited forms of hNEIL1 against urea lesions was also of interest, as Tg is repaired differentially by the two isoforms of hNEIL1 (34,43,46,47). Here, utilizing oligodeoxynucleotides containing site-specific urea lesions, we examine hNEIL1-initiated catalysis through a combination of biochemical studies and structural analyses of the urea lesions in DNA and in complex with the active-site mutant CΔ100 P2G hNEIL1 (K242) glycosylase.

## MATERIALS AND METHODS

### Site-specific urea lesions

- (a) 5′-CGTCCAXGTCTAC-3′

The oligodeoxynucleotide 5′-CGTCCAXGTCTAC-3′ (X = Tg) was purchased from the Midland Certified Reagent Co. (Midland, TX). The oligodeoxynucleotide 5′-TAGACATGGACGG-3′ was purchased from Integrated DNA Technologies, Inc. (Coralville, IA). The annealed ds DNA possessed a dangling 3′-C in the modified strand and a dangling 3′-G in the complementary strand. Both oligodeoxynucleotides were purified by reverse-phase HPLC using a Gemini C_18_ 250 mm × 10 mm column (Phenomenex, Inc., Torrance, CA). To prepare site-specific urea adducts, 80 nmol of the Tg-containing oligodeoxynucleotide were hydrolyzed overnight in 0.2 M sodium phosphate (pH 12.0) (64,66,68) at room temperature. The product 5′-CGTCCAXGTCTAC-3′ (X = urea lesion) existed as an equilibrium mixture of two species. The two species could be separated by C_18_ HPLC at 2 ml/min using a 5–10% acetonitrile gradient in 0.1 M ammonium formate (pH 8.0), but subsequently underwent re-equilbration to the mixture (Supplementary Figure S1 in the Supplementary Data). The identity of the product was confirmed by MALDI-TOF mass spectrometry (for the urea-adducted 13-mer, theoretical mass, 3819.6; observed mass, 3818.4).

- (b) 5′-CXGA-3′

The tetramer 5′-CTGA-3′ (Midland Certified Reagent Co.), at 28 μM, was oxidized with 1.4 mM KMnO_4_ in 0.2 M sodium phosphate (pH 8.6) for 5 min. The reaction was quenched by addition of sodium bisulfite (64,66,68,69). The mixture was centrifuged, filtered, and purified by HPLC using a Phenomenex Gemini 5 μm C_18_ 110 Å column in 0.1 M ammonium formate (pH 7.0). The flow rate was 2 mL/min. The gradient was 1% acetonitrile for 5 min, a linear gradient to 8% for 35 min, a linear gradient to 1% for 2 min, and 1% for 15 min. The major peak isolated was 5′-CXGA-3′, X = Tg. Its mass was confirmed by MALDI-TOF mass spectrometry. The Tg-modified tetramer was hydrolyzed in 0.025 M sodium phosphate (pH 12.0) overnight to quantitatively form the lesion 5′-CXGA-3′, X = urea lesion (64,66,69). It was purified by HPLC using a Phenomenex Gemini 5 μm C_18_ 110 Å column in 0.1 M ammonium formate (pH 7.0). The flow rate was 2 ml/min. The gradient was 1% acetonitrile for 5 min, a linear gradient to 8% for 35 min, a linear gradient to 1% for 2 min, and 1% for 15 min. The product 5′-CXGA-3′, (X = urea lesion) existed as an equilibrium mixture of two species. These could be separated by HPLC (Supplementary Figure S2 in the Supplementary Data) but subsequently re-equilibrated. The identity of the product was confirmed by MALDI-TOF mass spectrometry.

### Expression constructs

The construction of pET22b(+) vectors for expression of His-tagged edited (R242) and unedited (K242) versions of wild-type (wt) hNEIL1 was as previously described (31,43). To make a construct for the truncated hNEIL1 CΔ100 (K242) glycosylase, the corresponding pET22b(+) plasmid (with a C-terminal His-tag and an ampicillin resistance gene) that contained the full length *hNEIL1* cDNA sequence was used. The desired truncation was introduced using primers 5′-GAGGGGTGCCAACGGTCCAGGATC-3′ and 5′-GAGCACCACCACCACCACCACTGA-3′ (Integrated DNA Technologies, Inc., Coralville, IA) and Q5 site-directed mutagenesis kit (New England Biolabs Inc., Ipswich, MA). The P2G point mutation was introduced using primers 5′-GAGATATACATATGGGTGAGGGCCCCGAGCTGC-3′ and 5′-GTGCAGCTCGGGGCCCTCACCCATATGTATATCTC-3′ (Integrated DNA Technologies, Inc., Coralville, IA) and a site-directed mutagenesis kit (Agilent Technologies, Santa Clara, CA).

### Expression and purification of hNEIL1 glycosylases

Unedited (K242) and edited (R242) forms of the wt NEIL1 glycosylase were expressed and purified according to published protocols (31,41). To express the P2G CΔ100 hNEIL1 (K242) glycosylase, the corresponding plasmid was co-transformed with a chaperone expression vector, pGRO12 (containing a kanamycin resistance gene) into *E. coli* BL21 gold (DE3) competent cells (Agilent Technologies). The bacterial culture was grown at 37°C in Luria broth medium (Research Product International (RPI), Mt Prospect, IL) with 100 μg/ml ampicillin (RPI) and 50 μg/ml of kanamycin sulfate (RPI) until the OD_600 nm_ reached ∼0.6. The 0.01% arabinose was added to induce GroEL expression and after incubation of the cell culture at 18°C on a shaker for 2 h, 0.5 mM IPTG was added to induce expression of P2G hNEIL1 (K242) protein. Following a 22 h incubation at 18°C, cells were harvested and lysed using LM20 microfluidizer (Microfluidics, Westwood, MA) at 18000 psi in buffer containing 50 mM sodium phosphate (pH 7.5), 300 mM NaCl, 20% glycerol, 10 mM imidazole, 5 mM β-mercaptoethanol and protease inhibitor cocktail (Roche, South San Francisco, CA). Cell debris were removed by centrifugation at 18 000 rpm for 45 min at 4°C. After passage through a 0.45 μm filter, the supernatant was loaded onto a Ni-NTA Histrap HP 5 ml column (Cytiva Life Sciences, Marlborough, MA) using AKTA pure 25M (Cytiva) to bind the recombinant protein. The column was washed with ∼300 ml 50 mM sodium phosphate (pH 7.5) buffer, containing 300 mM NaCl, 20% glycerol, 40 mM imidazole and 5 mM β-mercaptoethanol, and the glycosylase was eluted in 50 mM sodium phosphate, (pH 7.5), 300 mM NaCl, 20% glycerol, 350 mM imidazole and 5 mM β-mercaptoethanol. The glycosylase was further purified by size exclusion, Superdex 200 increase (10/300 GL) (Cytiva) with 20 mM HEPES (pH 7.5) buffer, containing 100 mM NaCl, 10% glycerol and 2 mM Tris(2-carboxyethyl) hydroxy phosphine hydrochloride (TCEP). The purity was analyzed on 4–12% SDS PAGE. The mass was confirmed by MALDI-TOF mass spectrometry. The theoretical and observed masses were 33723.6 and 33570.18 Da, respectively. Mass analysis of the mutant glycosylase, following partial proteolysis, was used to detect contamination by host glycosylases. Protein concentration was determined by UV absorbance at 280 nm (*A*_280_, 1 mg/ml ∼0.97). For long-term storage, the protein was flash frozen in liquid nitrogen and stored at –80°C. The APE1 endonuclease was purchased commercially (New England Biolabs Inc., Ipswich, MA). The Fpg glycosylase was expressed and purified according to a published protocol (70).

### Glycosylase assays

Rate constants of hNEIL1-catalyzed base excision reactions were measured using ds 13-mer DNA substrates that contained a centrally located urea or Tg lesion and ^32^P at the 5′ terminus of the lesion-containing strand (5′-[^32^P]-CGTCCAXGTCTAC-3′ annealed with 5′-TAGACATGGACGG-3′; X = Tg or urea lesion). The conditions for the oligodeoxynucleotide labelling using γ-[^32^P]-ATP and the DNA strand annealing reactions were as previously described (39). The reaction rates were measured under single-turnover conditions following an established protocol (39). The reactions were conducted in 20 mM Tris–HCl (pH 7.4), 100 mM KCl, 100 μg/ml BSA, and 0.01% (v/v) Tween-20, at either 22 or 37°C. The DNA substrate and enzyme were separately pre-incubated for 3 min at the reaction temperature and combined at final concentrations of 20 nM and 1 μM, respectively. Aliquots were removed at selected time points, transferred to tubes that contained an equal volume of 0.5 M NaOH, and incubated at 90°C for 2 min. The oligodeoxynucleotides were resolved by electrophoresis in a 15% denaturing polyacrylamide gel containing 8 M urea and visualized using a Personal Molecular Imager (Bio-Rad Laboratories, Hercules, CA). The intensities of substrate and product bands were quantified by Quantity One software (Bio-Rad). The percent of product (*P*) was plotted as a function of time (*t*) and the rate constant (*k*_obs_), nonspecific product (*P*_ns_), and extrapolated maximal substrate utilization (*S*) were obtained by fitting the data to a single exponential equation *P* = *P*_ns_ + *S*(1 – exp^−*k*obs*^^t^) using KaleidaGraph 4.1 software (Synergy Software, Reading, PA). The mean *k*_obs_ values with standard deviations were calculated from a minimum of three repeats of the glycosylase activity assay.

The substrate specificity of CΔ100 P2G hNEIL1 (K242) mutant was also assessed using 17-mer ds oligodeoxynucleotides that contained either a site-specific Tg lesion, a natural AP site, or an AP site analog, THF. The substrate strands contained a carboxytetramethylrhodamine (TAMRA) moiety on the 5′ terminus (5′-TAMRA-TCACCTXCGTACGACTC-3′; X = Tg and 5′-TAMRA-TCACCXTCGTACGACTC-3′; X = AP site or THF). These substrate strands were annealed to a complementary strand that had a Black Hole Quencher 2 (BHQ2) on its 3′ terminus (5′-GAGTCGTACGAYGGTGA-BHQ2-3′; Y = A or C). The TAMRA-conjugated oligodeoxynucleotide containing Tg was previously described (71). The TAMRA-conjugated oligodeoxynucleotides containing uracil (AP site precursor) or THF and BHQ2-conjugated oligodeoxynucleotides were purchased from Integrated DNA Technologies, Inc. (Coralville, IA). The DNA substrate preparation and the experimental approach for measurement of hNEIL1 activity using TAMRA-conjugated oligodeoxynucleotides were previously reported (71). The reactions for detection of glycosylase and AP lyase activities were conducted in 20 mM Tris–HCl (pH 7.4), 100 mM KCl, 100 μg/ml BSA, and 0.01% (v/v) Tween 20 with 50 nM DNA substrate using enzyme concentrations as indicated in the corresponding figures. The reactions for detection of endonuclease activity were conducted in 20 mM Tris-acetate (pH 7.9), 50 mM potassium acetate, 10 mM magnesium acetate, and 100 μg/ml BSA (CutSmart buffer from New England Biolabs Inc.). The reaction volumes were 20 μl. The DNA substrate was aliquoted into wells of a black 384-well plate, reactions initiated by the addition of enzyme, and the TAMRA fluorescent signal was recorded in a TECAN plate reader at 37°C using a 525/9 nm excitation filter and a 598/20 nm emission filter.

### Nuclear magnetic resonance

Samples were in 600 μl of 100 mM NaCl, 50 mM Na_2_EDTA in 10 mM sodium phosphate (pH 7.0). To study non-exchangeable protons, samples were prepared in 99.996% D_2_O. To study exchangeable protons, samples were prepared in 95:5 H_2_O:D_2_O. Spectra were referenced to the chemical shift of water at the corresponding temperature, which was referenced to trimethylsilyl propanoic acid. Spectrometers operating at 500, 600, 800 and 900 MHz were used. Data processing used the program TOPSPIN (Bruker Biospin, Billerica, MA). Data were analyzed using the program SPARKY (72).

Correlated spectroscopy (COSY) data were collected with 2048 data points in the t_2_ dimension and 512 data points in the *t*_1_ dimension. The data were zero-filled to obtain matrices of 2048 × 1024 data points. Water suppression was accomplished by pre-saturation. Total correlated spectroscopy (TOCSY) spectra (73) were recorded with 2048 data points in the *t*_2_ dimension and 512 data points in the *t*_1_ dimension. The data were zero-filled to obtain final matrices of 2048 × 1024 data points. TOCSY spectra were collected in both D_2_O and H_2_O with a spin lock time of 120 ms. Water suppression in D_2_O was accomplished by pre-saturation, whereas water suppression in H_2_O was accomplished by excitation sculpting (74,75).

To observe non-exchangeable protons, NOESY spectra (76) were recorded in D_2_O with 2048 data points in the *t*_2_ dimension and 512 data points in the *t*_1_ dimension. The data were zero-filled to obtain final matrices of 2048 × 1024 data points. Data were collected at mixing times of 250 ms and 1 s. A relaxation delay of 2.0 s was used. States-TPPI quadrature detection was used. Water suppression was achieved by pre-saturation. To observe exchangeable protons, NOESY spectra in 95:5 H_2_O:D_2_O were collected with 2048 data points in the t_2_ dimension and 512 data points in the t_1_ dimension. The data were zero-filled to obtain final matrices of 2048 × 1024 data points. The mixing time was 200 ms. Water suppression was accomplished by excitation sculpting (74,75). Data were collected both at 274 and 278 K.

### Crystallization of CΔ100 P2G hNEIL1 (K242) with ds DNA containing a urea lesion

Purified CΔ100 P2G hNEIL1 (K242) (Supplementary Figure S3 in the Supplementary Data) was exchanged with 100 mM NaCl and 2 mM TCEP (no glycerol) in 20 mM HEPES (pH 7.5) and concentrated to 5 mg/ml using 10 kDa ultracentrifuge filters (Millipore Sigma, Burlington, MA). Oligodeoxynucleotides containing site-specific urea lesions were combined with stoichiometric amounts of the complementary oligodeoxynucleotides in 10 mM Tris–HCl (pH 8.0) containing 100 mM NaCl and annealed by heating to 85°C for 5 min followed by cooling to room temperature. The CΔ100 P2G hNEIL1 (K242) glycosylase and the urea-modified ds DNAs were combined at 1:1.2 molar ratio in 200 μl and incubated for 30 min at 0°C before passing over a size exclusion Superdex 200 (10/300GL) column pre-equilibrated with 100 mM NaCl and 2 mM TCEP in 20 mM HEPES (pH 7.5). Protein-DNA complexes having 1:1 stoichiometry were isolated, concentrated to 4 mg/ml, and filtered through 0.22 μm filters. Initial crystallization conditions were established with the 96-well Natrix screen (Hampton Research, Aliso Viejo, CA) at 14°C from fresh preparations of protein–DNA complexes using the sitting drop vapor diffusion technique. Screening hits were then expanded for obtaining diffraction quality crystals. Rod-shaped crystals were formed by combining 1 μl of protein–DNA complex and a reservoir containing 0.01 M magnesium sulfate and 1.8 M lithium sulfate in 0.05 M sodium cacodylate (pH 6.0). After 2 weeks, crystals were transferred into a reservoir containing 20% ethylene glycol as cryoprotectant and flash frozen in liquid nitrogen.

### Crystallographic data collection and structure refinement

Diffraction data were collected on beamline 21-ID-G at LS-CAT, Advanced Photon Source (APS, Argonne National Laboratory, Argonne, IL) at a wavelength 0.97856 Å. Initial data processing, including integration and scaling, were done using the software package HKL2000 (77) (HKL Research, Inc., Charlottesville, VA). The structure was determined by molecular replacement with Phaser (78) using the structure of the hNEIL1-Tg modified ds DNA (PDB ID: 5ITX) as a search model (47). Initial rounds of rigid body refinement were followed by restrained refinement using REFMAC5 (79). Further refinement and model building were performed with Phenix (80) and Coot (81). PROCHECK (82) was used to assess the quality of final refined model. Final data collection and structure refinement parameters are listed in Table 1. Structure figures were prepared in PyMOL (The PyMOL Molecular Graphics System, Version 2.5.2, Schrödinger, LLC, New York, NY). Protein-DNA interactions were generated using PDBsum server (83).

**Table 1.** Crystal data, X-ray data collection, and refinement statistics

	[CΔ100 P2G hNEIL1 (K242)]- Urea Lesion-dsDNA	
**PDB entry**	8FTJ	
**SBGrid entry**	909	
**X-ray Data collection**		
X-ray source	APS LS-CAT, 21-ID-G	
Wavelength [Å]	0.97856	
Space group	*P* 4_1_	*P* 4_1_ 2 2
Unit cell		
*a* [Å]	83.66	83.66
*b* [Å]	83.66	83.66
c [Å]	130.44	130.44
α/*β*/*γ* [°]	90, 90, 90	90, 90, 90
Resolution [Å]	50 - 2.3 (outermost shell: 2.34–2.30)	50 - 2.3 (outermost shell: 2.34–2.30)
Reflections	39805	21210
*R*_sym_	0.21(1.8)	0.23 (1.5)
*R*_pim_	0.08 (0.6)	0.06 (0.4)
*I*/σ(*I*)	12.3 (1.5)	16.1 (1.94)
Completeness [%]	99.5 (99.1)	99.3 (98.7)
Redundancy	8.3 (8.3)	15.5 (14.6)
CC_1/2_	0.994 (0.61)	0.985 (0.72)
**Refinement**		
No. of molecules per asymmetric unit	2	1
*R*_cryst_ [%]	19.9	20.0
*R*_free_ [%]	24.9	24.3
RMS deviation		
bond length [Å]	0.008	0.008
bond angles [^o^]	1.00	0.98
Ramachandran [%] (PROCHECK)		
favoured	90.0	90.1
allowed	10	9.4
generous	0.0	0.5
outliers	0.0	0.0
*B*-factor [Å^2^]	29.7	30.5
No. of atoms	5420	2796
No. of residues		
Protein	524 (Chain A–B)	263 (Chain A)
DNA	52 (Chain C–D)	26 (Chain D)
Water	222	127

## RESULTS

### Anomeric interconversion of urea lesions

**Figure 1.**
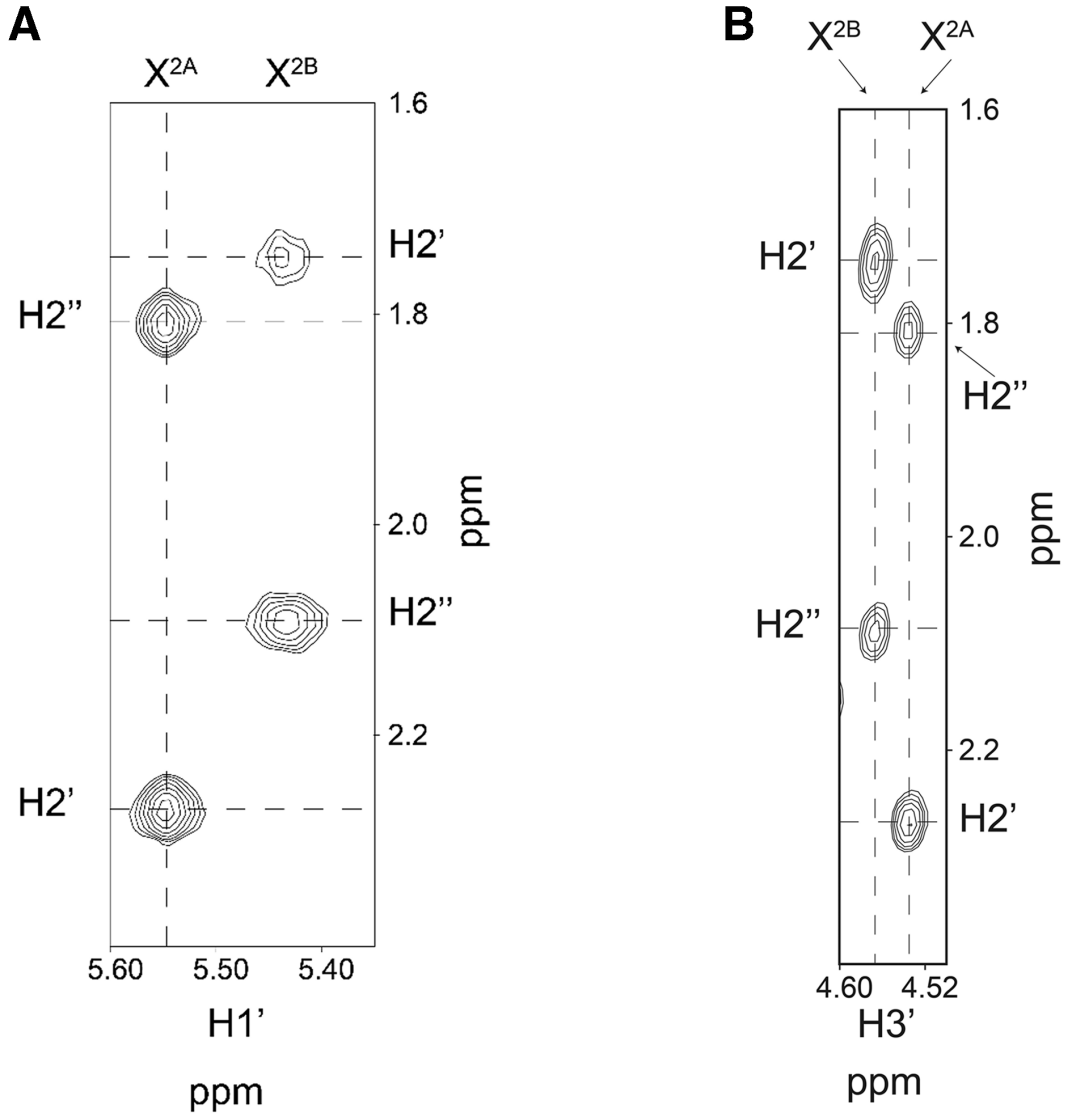
NMR characterization of the urea lesion anomers. Two deoxyribose H1′ resonances were assigned from NOESY and TOCSY spectra. These were attributed to the urea lesion due to the absences of the resonances in the pyrimidine H6 and purine H8 and H1′ region of the spectrum. (**A**) A NOESY experiment with a mixing time of 250 ms established that for the β anomer, the NOE between the H2″ and H1′ protons was stronger than the NOE between the H2′ and H1′ protons. For the α anomer, the NOE between the H2′ and H1′ protons was stronger than the NOE between the H2″ and H1′ protons. (**B**) For both anomers, the NOEs between the H2′ and H3′ protons were stronger than were the NOEs between the H2″ and H3′ protons.

Prior studies of the structure of the urea nucleoside (66,67) revealed the existence of two species in slow exchange, ascribed to α and β anomers of the 2′-deoxyribosyl ring (67) (Scheme 1B). Prior investigations in DNA had also revealed two species in slow exchange, but these had not been interpreted unequivocally (84). To determine if the anomeric interconversion reported at the nucleoside level (67) persisted in DNA, we conducted HPLC analyses of urea lesions in 5′-CGTCCAXGTCTAC-3′ and in 5′-CXGA-3′ (X = urea lesion) (Supplementary Figures S1 and S2 in the Supplementary Data). These analyses revealed two chromatographically separable peaks in significant amounts. Re-injection of each peak individually yielded the original chromatogram, indicating that following the isolation of each peak, re-equilibration occurred. The re-equilibration was more rapid at pH <7, consistent with acid-catalyzed interconversion between two anomers. NMR studies in 5′-CXGA-3′ (X = urea lesion) revealed two deoxyribose H1′ resonances at the lesion site, shifted upfield 0.2 ppm relative to H1′ resonances of the non-modified nucleotides (Figure 1). Each exhibited an integrated intensity approximately one-half that of the H1′ resonances arising from unmodified nucleotides, consistent with HPLC analyses. The anomeric configurations were established from the respective magnitudes of the NOEs between each H1′ proton and the corresponding deoxyribose H2′ and H2″ protons (Figure 1). In addition, the TOCSY spectrum revealed correlations between the H1′ protons and the corresponding amine protons of the urea lesion. The ^3^*J* couplings to the H1′ protons were 9.0 and 8.7 Hz, respectively (Supplementary Figure S4 in the Supplementary Data). For both anomers, the deoxyribose H1′ and amine protons were in the *trans* conformation. NMR studies in the longer oligodeoxynucleotide 5′-CGTCCAXGTCTAC-3′ (X = urea lesion), which was used for subsequent biochemical and crystallographic analyses, were inconclusive due to the complexity of the NMR data. It was not possible to unequivocally identify which HPLC chromatographic peak corresponded to which anomeric form of the urea lesion.

### Efficient incision of DNA containing urea lesions by edited and unedited hNEIL1

**Figure 2.**
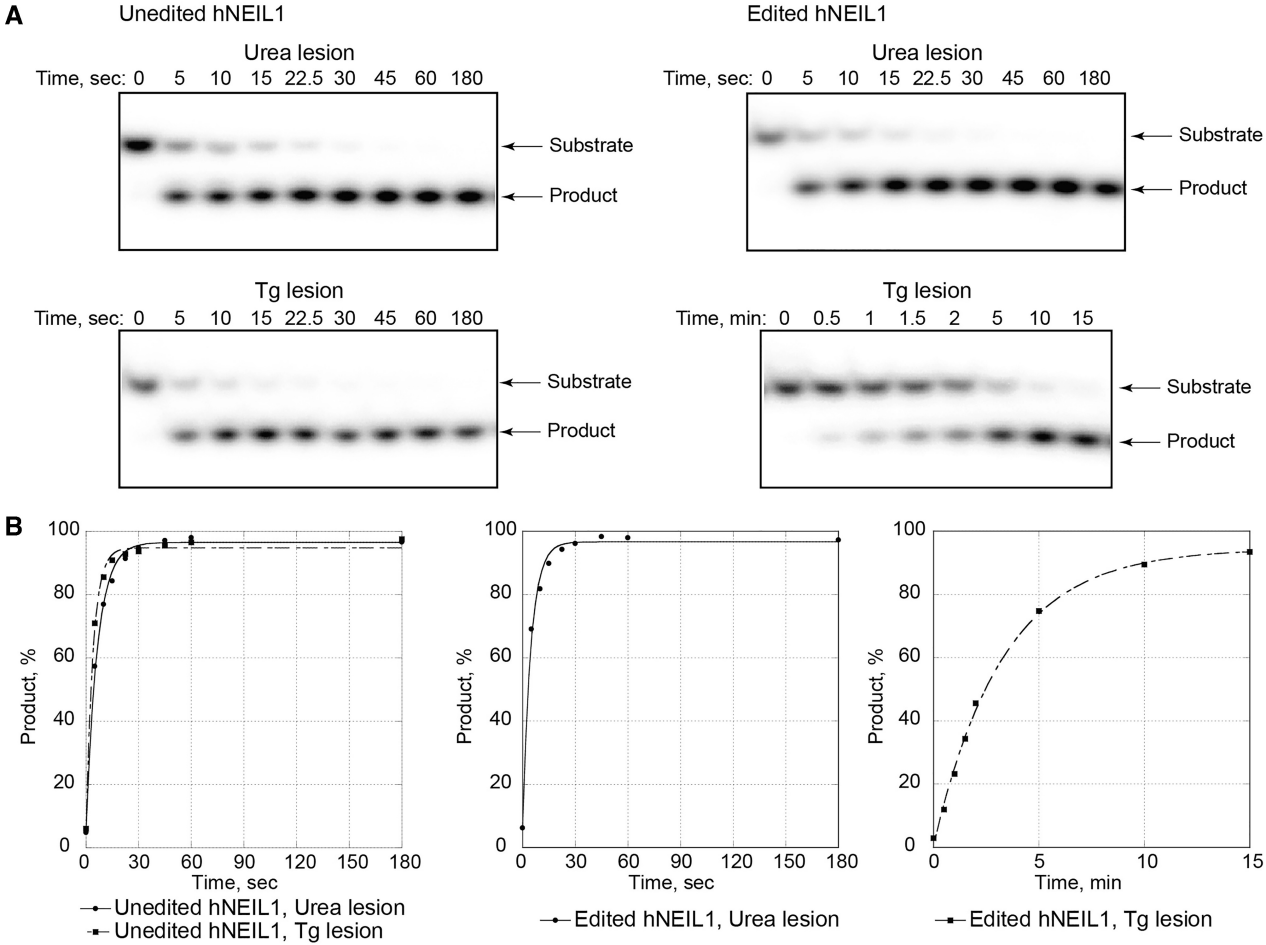
Excision of urea and Tg lesions by hNEIL1. Reactions were conducted under single-turnover conditions at 22°C. DNA substrates were ds 13-mer oligodeoxynucleotides that contained a centrally located urea or Tg lesion and were labelled with ^32^P at the 5′ ends of the lesion-containing strands. Representative gel images (**A**) and corresponding plots (**B**) demonstrating the time-dependent product accumulation. Experimental data could be fit to a single exponential kinetic curve (*R*^2^ > 0.99). The mean *k*_obs_ values with standard deviations reported in Table 2 were calculated from a minimum of three repeats of the glycosylase activity assay.

Analyses of hNEIL1 glycosylase activity utilized the ds 5′-CGTCCAXGTCTAC-3′:5′-TAGACATGGACGG-3′, X = urea lesion. Following separation by HPLC (Supplementary Figure S1 in the Supplementary Data), the adducted oligodeoxynucleotides that contained anomers of the urea lesion were individually labelled with ^32^P at the 5′ termini and annealed with the complementary strand. Initial experiments assessing the ability of hNEIL1 to excise the urea lesion were carried out with the edited form of hNEIL1 (R242) using 37.5-fold molar enzyme excess over DNA. The reactions were incubated at 37°C and product formation measured at 5 and 30 min. These data revealed the complete incision of both urea-containing substrates by the edited hNEIL1 (R242) in 5 min. DNA with an identical sequence that contained a site-specific Tg lesion was also prepared, and experiments were conducted to determine the relative rate constants for excision of the urea *vs*. Tg lesions by the unedited (K242) and edited forms (R242) of hNEIL1 under comparable conditions (Figure 2). Due to the rapid rates of incision, these reactions were incubated at 22°C as opposed to 37°C. Following incubation, DNA was immediately treated with 250 mM NaOH to convert the products of base excision, AP sites, into DNA strand breaks. Quenching reactions with NaOH allowed for measurement of hNEIL1 glycosylase activity separately from its lyase activity. On substrates containing the urea lesion, the unedited and edited forms of hNEIL1 exhibited comparable efficiencies (Table 2 and Figure 2). We observed no differences in rates of excision between urea-containing substrates initially prepared from either oligodeoxynucleotide, as separated by HPLC (Supplementary Figure S1 in the Supplementary Data). In contrast and consistent with prior reports (34,43,46,47), excision of Tg by the unedited enzyme was ∼ 50-fold more efficient than by the edited hNEIL1 (Table 2 and Figure 2). For the unedited hNEIL1, rates of excision of the urea lesion were ∼30% lower relative to that of Tg. However, they exceeded by >30-fold the rate of excision of Tg by the edited hNEIL1.

**Table 2.** Excision rate constants (*k*_obs_) for Tg and urea adducts by unedited and edited hNEIL1

Enzyme	Unedited hNEIL1 (K242)		Edited hNEIL1 (R242)	
Substrate	Urea	Tg	Urea	Tg
*k*_obs_, min^−1^ (AVG ± STDEV)	11 ± 3	16 ± 1	11 ± 2	0.32 ± 0.02

### The CΔ100 P2G hNEIL1 (K242) glycosylase complexed with DNA containing a urea lesion

**Figure 3.**
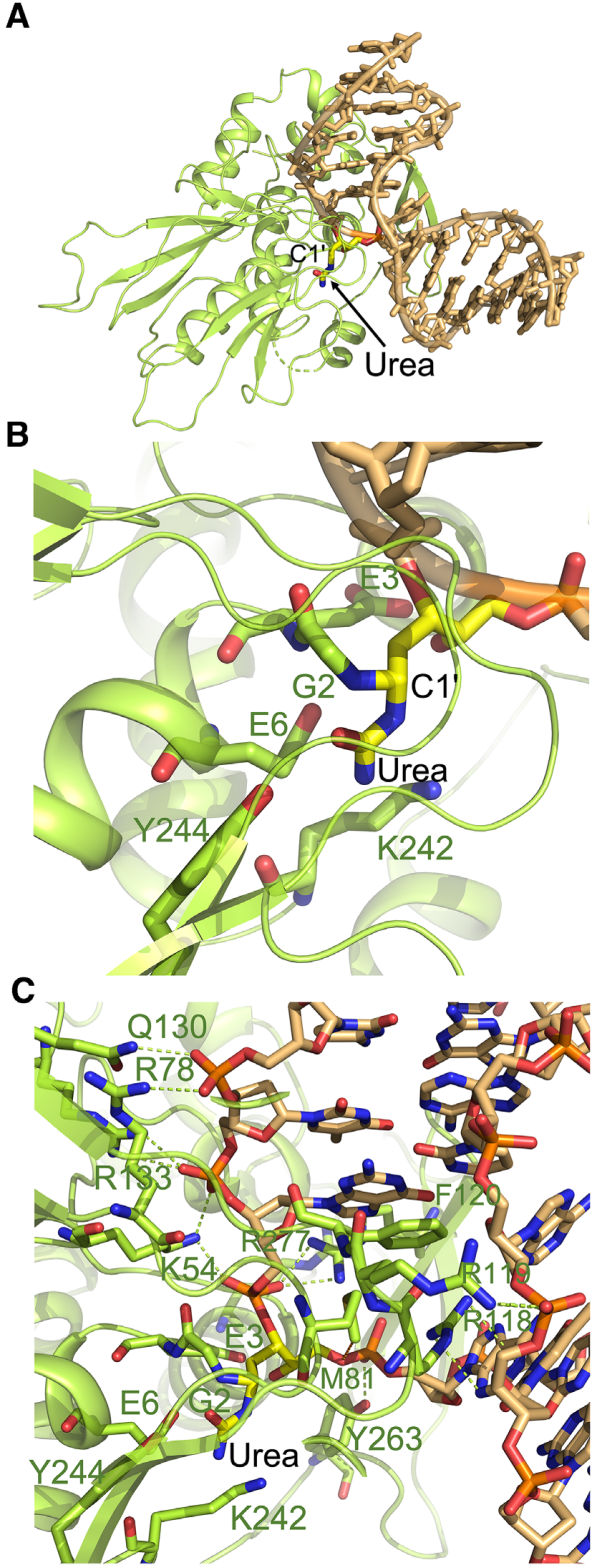
Crystal structure of CΔ100 P2G hNEIL1 (K242) bound to the urea-containing ds DNA. (**A**) Overall view of the monomeric hNEIL1 protein (green) bound to the DNA, with the C1′ atom of the flipped ring-opened deoxyribose covalently linked to the urea lesion (yellow). (**B**) Protein residues Gly2, Glu3, Glu6, Lys242 and the flipped nucleotide (with intact urea moiety) at the active site. (**C**) View of the extruded urea nucleotide lodged in the active-site pocket with residues Met81, Arg118 and Phe120 filling the void in the ds DNA. Protein residues Lys54, Arg78, Gln130, Arg133, Arg118, Arg119, R277 and Tyr263 make H-bonded contacts with DNA backbone atoms.

**Figure 4.**
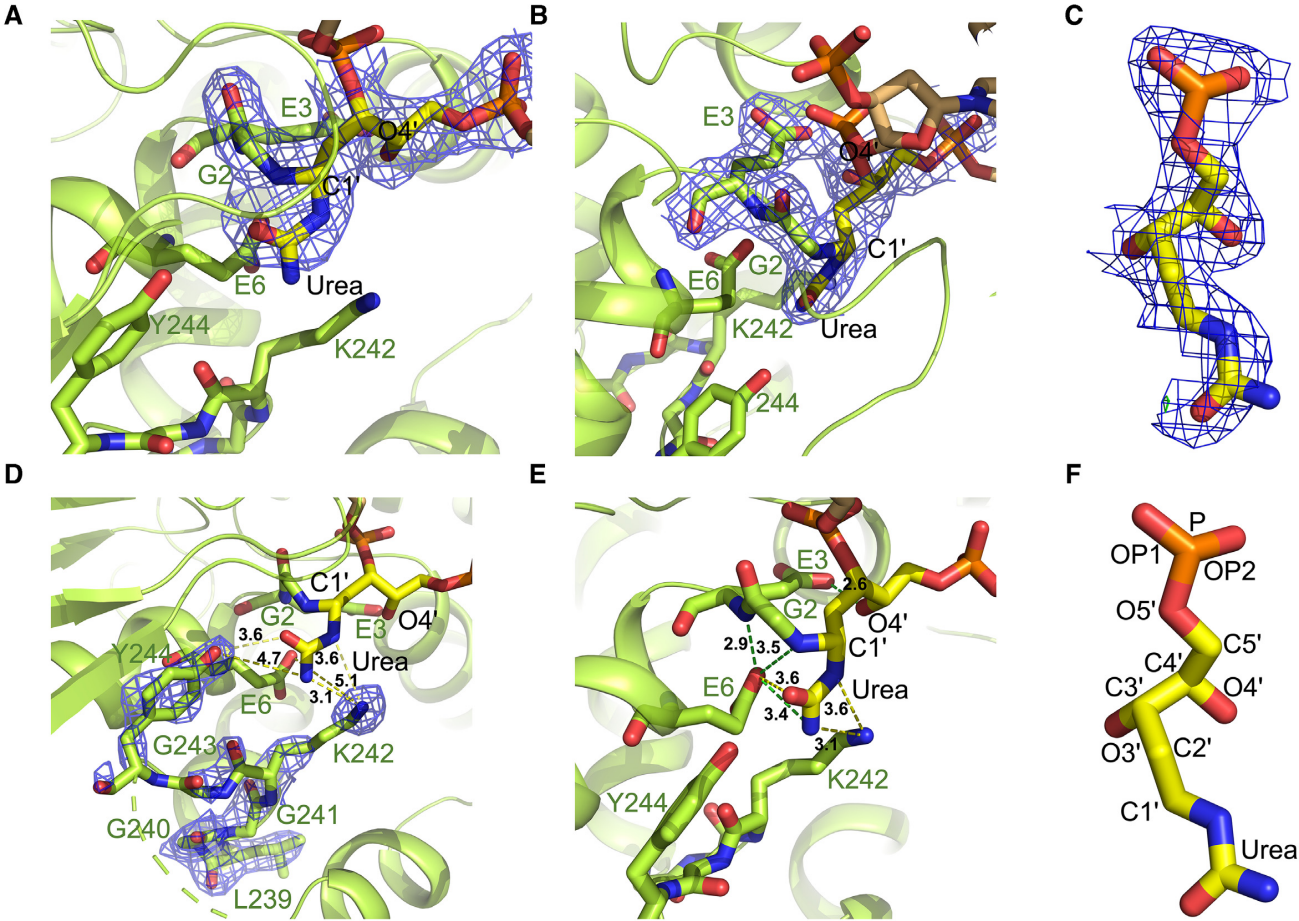
Active-site interactions in the complex between CΔ100 P2G hNEIL1 (K242) and urea lesion-containing DNA. (**A**) 2Fo-Fc electron density maps (contoured at 1σ) (dark blue mesh) of protein residue Gly2 (green) and flipped nucleotide (yellow) at the active site. Contiguous electron density between flipped nucleotide and the Gly2 residue was observed at the point of interaction between Gly2 nitrogen and C1′ of the ring-opened deoxyribose. (**B**) 2Fo-Fc electron density maps (contoured at 1σ) (dark blue mesh) at the active site, of protein residues Gly2, Glu3, and the flipped nucleotide. Contiguous electron density between Glu3 acidic chain and deoxyribose O4′ was evident. (**C**) 2Fo-Fc electron density maps (contoured at 1σ) (dark blue mesh) of the expanded flipped nucleotide. (**D**) 2Fo-Fc electron density maps (contoured at 1σ) (dark blue mesh) of loop residues (green) Gly240, Gly241, Lys242, Gly243 and Tyr244 near the active-site are part of a flexible region. (**E**) H-bonded interactions (green dotted lines) between protein residues (green) and flipped nucleotide (yellow) and the associated distances (yellow dotted lines) are shown at the active site. The ring-opened deoxyribose was fused with the urea chain at C1′ and the Gly2 α-amine was covalently linked to deoxyribose C1′. The deoxyribose sugar O4′ was H-bonded with Glu3. Glu6 also H-bonded with the Gly2 α-amine, the Gly2-Glu3 amide, and the urea amine groups. Flexible loop residue Lys242 was within H-bonding distance from the urea amine. (**F**) Expanded structure of the ring-opened deoxyribose atoms covalently linked to the urea chain. All H-bonded (green dotted lines) and non-bonded (yellow dotted lines) distances highlighted as numbers are in [Å].

**Scheme 2.**
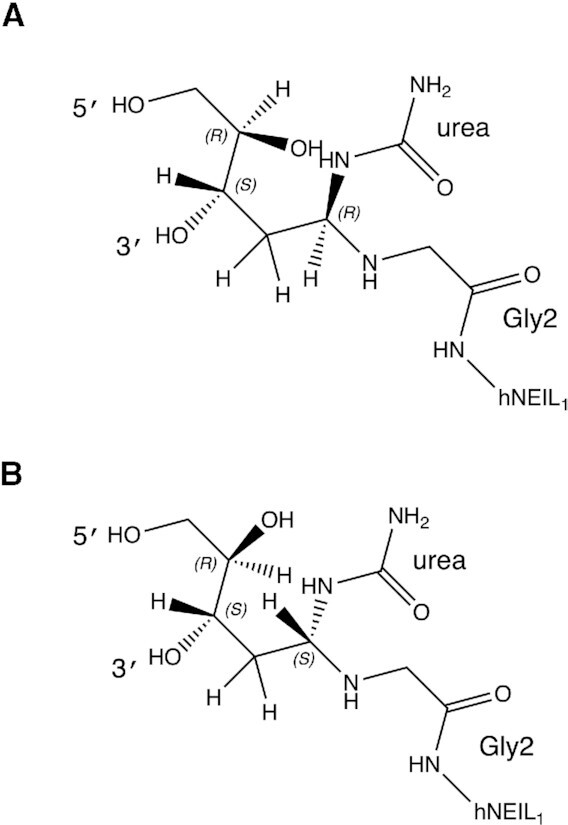
The ring-opened deoxyribose configuration of the flipped nucleotide. (**A**) *R* diastereomer following attack of N-terminal amine of Gly2 on the anomeric carbon at the urea lesion. (**B**) *S* diastereomer following attack of N-terminal amine of Gly2 on the anomeric carbon at the urea lesion.

The active site hNEIL1 mutant, CΔ100 P2G hNEIL1 (K242), was used to investigate the structural basis of urea excision. The oligodeoxynucleotide 5′-d(CGTCCAXGTCTAC)-3′:5′-d(TAGACATGGACGG)-3′, X = urea lesion, was separated into anomeric species by HPLC (Supplementary Figure S1 in the Supplementary Data). DNA from each of the two anomeric species was annealed with the complementary oligodeoxynucleotide. These ds DNAs were then individually combined with CΔ100 P2G hNEIL1 (K242) to form protein–DNA complexes and subjected to crystallization trials. The complex that was prepared from DNA in the first eluting HPLC peak (Supplementary Figure S1 in the Supplementary Data) afforded a crystal that diffracted at 2.3 Å resolution. This crystal was used for subsequent analyses. This structure of CΔ100 P2G hNEIL1 (K242) in complex with the urea-containing ds DNA was determined in space groups *P*4_1_ and *P*4_1_22 at 2.3 Å resolution through molecular replacement using the previously published structure with PDB ID CODE 5ITX as search model (47). A comparison of data processing and refinement parameters is shown in Table 1. The structure emergent from the refinement in the *P*4_1_22 space group exhibited improved electron density for the loop residue Tyr244, as compared to the refinement in the *P*4_1_ space group (Supplementary Figure S5 in the Supplementary Data). Therefore, it was chosen for further analyses. During protein expression in *E. coli*, the N-terminal Met1 residue is susceptible to cleavage by methionine aminopeptidase (85,86). This residue was not observed in the final electron density maps and its absence was confirmed by mass spectrometry. The N-terminal residue of the CΔ100 P2G hNEIL1 (K242) glycosylase in the crystal structure is Gly2. Examination of the structure revealed that the urea-modified nucleotide was extruded from the DNA and lodged in the nucleobase binding pocket employed by the glycosylase for substrate recognition and excision (Figure 3) (47). Continuous electron density was observed between the deoxyribose C1′ and the N-terminal amine of Gly2 (Figure 4). When single bond distance (1.47 Å) restraints between corresponding C and N atoms were provided, the refined structure fitted better into the electron density. This indicated a covalent linkage between the Gly2 N-terminal amine and the deoxyribose C1′. Analyses of Fo-Fc electron density maps adjacent to deoxyribose C1′ (Supplementary Figure S6 in the Supplementary Data) suggested that the *R* diastereomer was a better fit than was the *S* diastereomer (Scheme 2). Therefore, this complex likely arose from ds DNA containing the β-anomer of the urea lesion.

The 2Fo-Fc electron density maps indicated the ring-opened deoxyribose configuration of the flipped nucleotide (Figure 4 and Scheme 2). Glu3 established a 2.6 Å H-bond with deoxyribose O4′, consistent with its carboxylate delivering a proton to O4′ as appeared from the merged electron density between these (Figure 4). The urea lesion remained covalently linked to the deoxyribose C1′ atom (Figures 3 and 4). The carbonyl and the amine groups of the urea were within 3.6 and 3.4 Å of the Glu6 carboxylate, respectively (Figure 4). The latter was also within 2.9 Å of the Gly2–Glu3 peptide bond amide nitrogen, and within 3.5 Å of the Gly2 α-amino nitrogen. Most of the electron density for the substrate recognition loop, consisting of amino acid residues 240–252, was missing. However, residues Gly240, Gly241, Lys242 and Tyr244 exhibited partial electron density as indicated in 2Fo-Fc electron density maps (Figure 4). The Fo-Fc electron density maps or omit maps (contoured at 3σ) also support the position of these residues (Supplementary Figure S5 in the Supplementary Data). The amino group of the flexible loop residue Lys242 was positioned at 3.1 and 3.6 Å from the amine and the imine of the urea moiety, respectively, suggesting the potential for H-bond formation (Figure 4). By comparison, loop residue Tyr244 was located farther away from urea moiety (Figure 4 and Supplementary Figure S5 in the Supplementary Data). It was not possible to distinguish between tautomers of the urea lesion (Scheme 1C) since at this resolution, both could be fit into the electron density (Supplementary Figure S7 in the Supplementary Data).

The void in the DNA helix created by the flipped nucleotide was stabilized by amino acids Met81, Arg118, and Phe120 (Figure 3). The Met81 methyl group was within van der Waals distance of the deoxyribose sugar O5′ of the urea lesion. The Arg118 guanidino moiety interacted with the 5′-neighboring (with respect to the urea lesion) adenine by establishing H-bonds with the adenine N3 atom. The Arg118 NH_2_ group formed an H-bond with the N3 atom of the adenine opposite to the damaged nucleotide in the complementary strand of DNA. The Arg118 also H-bonded with a water molecule, which via a second water molecule, was H-bonded to the phosphate group of the damaged nucleotide. The aromatic ring of Phe120 stacked over the 3′ neighbor G:C base pair (Figure 3). These interactions were similar to those observed in the co-crystal structures of hNEIL1 with DNA containing a non-excisable THF lesion or containing base modifications arising from oxidative damage (47,58). Several additional putative H-bonding interactions between protein residues and DNA are highlighted in Figure 3. All protein–DNA interactions generated from PDBsum server (83) are shown in the diagram (Supplementary Figure S8 in the Supplementary Data).

### Residual activity of the CΔ100 P2G hNEIL1 (K242) mutant glycosylase

**Figure 5.**
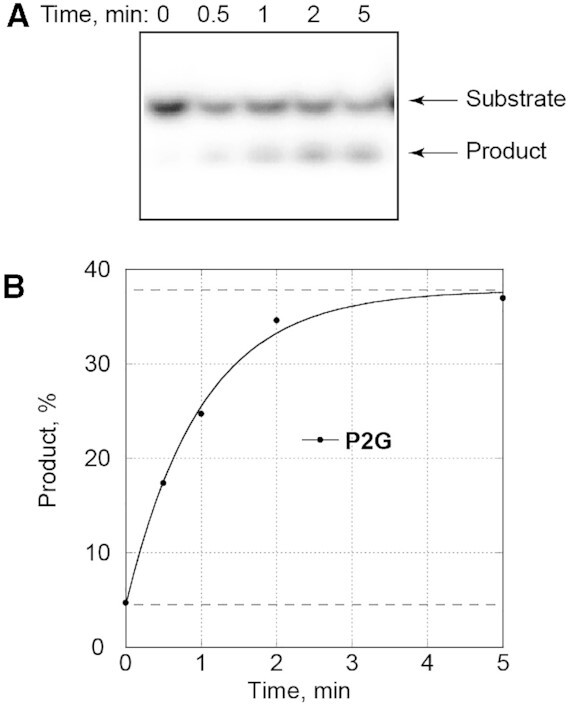
Glycosylase activity of CΔ100 P2G hNEIL1. Reactions were conducted under single-turnover conditions at 37°C. The DNA substrate was a ds 13-mer oligodeoxynucleotide that contained a centrally located urea lesion and was labelled with ^32^P at the 5′-end of the lesion-containing strand. A representative gel image (A) and the corresponding plot (**B**) demonstrating time-dependent product accumulation. Experimental data could be fit to a single exponential kinetic curve (*R*^2^ > 0.99). The lower dotted line corresponds to the percent of non-specific product that was calculated from a single exponential equation. The upper dotted line corresponds to combined fractions of non-specific product and the maximal substrate utilization. To obtain the mean values with standard deviations for *k*_obs_, non-specific product, and maximal substrate utilization, the glycosylase activity assay was repeated three times.

The observation of a pre-cleavage intermediate in the crystallographic data suggested that the CΔ100 P2G hNEIL1 (K242) glycosylase might retain residual activity for the urea lesion. Activity assays involving the mutant glycosylase were carried out in triplicate under the same conditions as for the wt hNEIL1, except that since the efficiency of the CΔ100 P2G hNEIL1 (K242) glycosylase was compromised, the temperature was increased to 37°C. These experiments were conducted using DNA containing HPLC-separated anomers of the urea lesion (Supplementary Figure S1 in the Supplementary Data). With the mutant glycosylase, excision products accumulated for ∼ 2 min, but plateaued (Figure 5). Approximately 30% of the initial DNA substrate molecules were incised. These results differed from those obtained for wt hNEIL1 glycosylase, which fully cleaved urea lesion-containing substrate (Figure 2). The inability of the mutant glycosylase to completely incise DNA containing the urea lesion was not explained by its inactivation under the conditions of reaction since addition of another aliquot of the glycosylase did not result in additional product formation.

To evaluate maximal substrate utilization and rate of excision of cleavable urea species, the data were fit to a single exponential (Figure 5). The mean values with standard deviations were 33.6 ± 4.2% and 0.86 ± 0.14 min^−1^, respectively. These did not differ depending on which anomeric fraction of the DNA was initially used to prepare the ds DNA substrates, likely due to subsequent re-equilibration of the anomers.

Following observation of incision by the CΔ100 P2G hNEIL1 (K242) glycosylase of urea-containing DNA, additional DNA substrates were examined (Supplementary Figure S9 in the Supplementary Data). The experimental design utilized 5′-TAMRA-conjugated lesion-containing oligodeoxynucleotides, annealed to complementary strands modified with BHQ2 at the 3′ terminus. DNA incision at the lesion site and subsequent dissociation of the TAMRA-conjugated product from the complementary strand would result in fluorescence enhancement that was monitored using a plate reader. Consistent with prior results (47), no nicking activity was observed on DNA containing the non-hydrolyzable AP site analog THF or the Tg lesion. However, the CΔ100 P2G hNEIL1 (K242) glycosylase exhibited a detectable concentration-dependent activity on DNA that contained an AP site opposite to A. The lyase activity of the CΔ100 P2G hNEIL1 (K242) glycosylase decreased when the AP site was located opposite C rather than A. Such a preference is like wt hNEIL1 and in contrast to *E. coli* Fpg. In agreement with proteomic mass spectrometric analyses, this series of experiments did not show a contamination of the CΔ100 P2G hNEIL1 (K242) glycosylase preparation by host AP endonucleases or glycosylases, such as Fpg, endonuclease III (Nth) or VIII (Nei). The two latter glycosylases efficiently excise Tg (27,87–89), but this was not observed in the presence of the CΔ100 P2G hNEIL1 (K242) glycosylase. Overall, these data demonstrate limited DNA glycosylase/AP lyase activity in the CΔ100 P2G hNEIL1 (K242).

## DISCUSSION

Urea lesions may arise as secondary products from Tg or 8-oxo-dG oxidative lesions in DNA. They have the potential to inhibit DNA replication and induce mutations (60,63–65). DNA polymerases that belong to the B or X families are blocked by urea lesions either immediately before the damage site (pols β and ϵ) or following preferential insertion of dAMP opposite to it (pols δ, α and ζ) (90). The Y family of DNA polymerases Pol η and Dpo4 bypass urea lesions, but with poor discrimination between incoming dNTPs (90). The mutagenic potential of the urea lesions is thought to be due to their ability to form H-bonding interactions with dAMP, dGMP and dTMP (91). Consequently, repair of urea lesions in DNA is of interest. They have been reported to be substrates for hNEIL1 (27). Urea lesions may also be repaired by the *E. coli* exonuclease III (61), *E.coli* endonucleases III (Nth) (27,62,68,87) and VIII (Nei) (27,88) and by the human homolog of *E. coli* endonuclease III (hNTH1) (27,92).

### The urea lesion equilibrates between α and β anomers in DNA

Urea lesions equilibrate between α and β deoxyribose anomers in DNA (Figure 1), as had been reported at the nucleoside level (67). A prior study of the urea lesion in ds DNA afforded NMR spectra indicating two species in slow exchange and proposed conformational isomerization about the peptide-like carbon-nitrogen bond of the urea moiety (84). While rotation about the carbon-nitrogen bond is anticipated, we surmise that the slowly exchanging species observed here are the two anomers. At neutral and basic pH, the interconversion between them is sufficiently slow to afford their separation by HPLC, but following isolation, re-equilibration to the mixture occurs sufficiently rapidly that it was not possible to conduct biochemical assays or crystallographic trials on anomerically-pure ds DNA substrates. The interconversion becomes more rapid at pH <7, consistent with the expectation that the anomeric interconversion is acid catalyzed. In the tetramer 5′-CXGA-3′ (X = urea lesion), the two anomers exist in approximately equal amounts (Figure 1). The complexity of the NMR spectra prevented quantitation of the anomeric ratio in ds DNA corresponding to our biochemical and crystallographic studies, but the HPLC separations suggest that significant quantities of both anomers are present at equilibrium under experimental conditions utilized for biochemical analyses and crystallization trials (Supplementary Figures S1 and S2 in the Supplementary Data).

### Excision of urea *vs*. Tg by unedited and edited hNEIL1

Urea lesions are removed by unedited and edited forms of hNEIL1 with comparable efficiencies (Figure 2 and Table 2). This contrasts with Tg and several other DNA substrates of hNEIL1 that are excised differentially by unedited vs. edited forms of the glycosylase (34,37,43,44,46,47,58). The rate of excision of urea lesions by the unedited hNEIL1 (K242) is 30% less than that of Tg, but excision rates by the edited hNEIL1 glycosylase (R242) are greater than that of Tg (Figure 2 and Table 2). This suggests that the transition state for excision of urea is not differentially affected by editing of the hNEIL1 glycosylase. The partial electron density of residue Lys242, as seen in the urea-bound complex structure (Figure 4) reveals some degree of disorder for this critical loop residue. It seems plausible that there may be intrinsically weaker interactions between urea lesions and residue 242 (either Lys242 or Arg242), which may not be rate-determining in the removal of the urea lesion. For Tg, it has been hypothesized that the greater rate of excision by the unedited hNEIL1 (K242) manifests a compromised substrate-checking mechanism, leading to erroneous excision of thymine (58). Calculations by Zhu *et al.* (47) supported H-bonding of Arg242 in the active site of the edited glycosylase. This might enhance selectivity for Tg versus thymine, at the expense of a less favourable transition state for Tg excision, compared to the unedited (K242) glycosylase. It has been proposed that the edited glycosylase provides a ‘slower but safer’ means of Tg excision (58). Lotsof *et al.* (46) evaluated isoform-specific repair on a series of oxidatively damaged bases. The unedited hNEIL1 glycosylase (K242) exhibited greater activity for oxidized pyrimidine lesions, including Tg, uracil glycol, 5-hydroxyuracil and 5-hydroxymethyluracil, with relative rates of excision by unedited versus edited form being correlated with the ability of these bases to form stable lactim tautomers and the affinity of their N3 atoms to protonation. The 5-hydroxycytosine and guanidinohydantoin bases were repaired more efficiently by the edited hNEIL1 glycosylase (R242), consistent with distinct chemical and structural features present in these lesions (46).

### The CΔ100 P2G hNEIL1 (K242) glycosylase in complex with urea-containing DNA

**Scheme 3.**
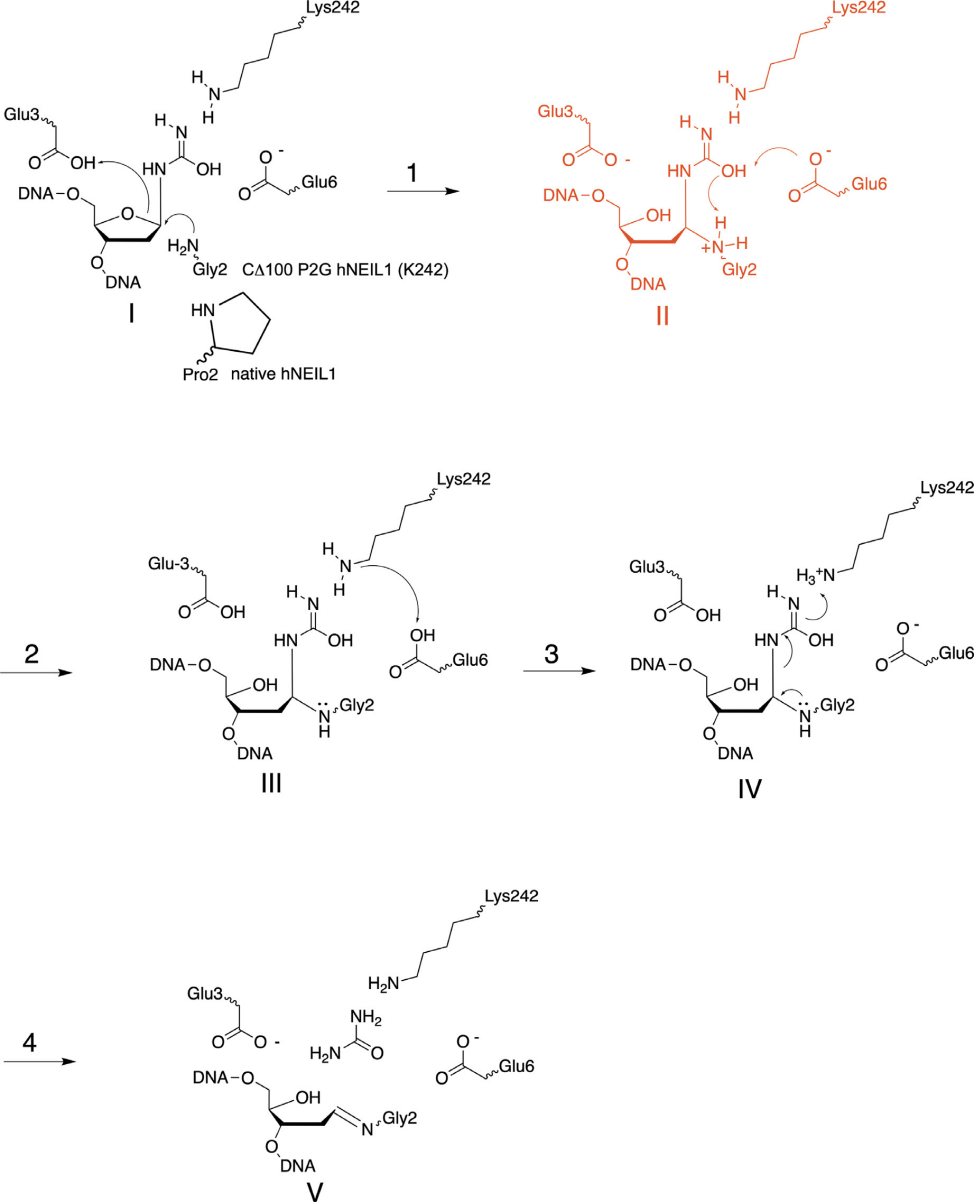
A reaction pathway, comparable to that proposed by Zhu *et al.* (47) for excision of the Tg lesion by the hNEIL1 DNA glycosylase. Substrate-recognition loop residue Lys242 interacts with the imino tautomer of urea lesion. In step 1, Glu3 facilitates attack by the N-terminal nucleophile amine of Gly2 in the mutant glycosylase or Pro2 in the native hNEIL1 at deoxyribose C1′. In step 2, Glu6 accepts a proton from the urea hydroxyl, which in turn accepts a proton from the protonated N-terminal nucleophilic amine. Species II (shown in red) is the ring-opened covalent intermediate trapped in the present crystal structure validating O4′ protonation by Glu3 carboxylate group. In step 3, Lys242 accepts a proton from Glu6. In step 4, re-arrangement of the N-terminal nucleophilic conjugate into an imine linkage results in the release of the urea lesion. Finally, the AP lyase activity of hNEIL1 catalyzes β- and β-δ elimination reactions to create a strand break (not shown).

In the pre-cleavage complex observed here, the N-terminal amine of Gly2 is conjugated to the deoxyribose C1′ and the flipped nucleotide appears in the ring-opened deoxyribose configuration with a covalently attached urea moiety (Figures 3 and 4). The proximity of O4′ to the carboxyl group of Glu3 is consistent with H-bonding. This illustrates the role of active site residue Glu3 in the hNEIL1 glycosylase in activating the C1′ and facilitating nucleophilic attack through protonation of deoxyribose O4′, consistent with the catalytic mechanism proposed (47,58) for Tg and other substrates (Scheme 3). This ‘ribose-protonated’ mechanism of base excision has been postulated previously (48) and proposed by Sadeghian *et al.* for the hOGG1 (93) and MutM glycosylases (94). The missing electron density for the loop region, residues 240–252, in the CΔ100 P2G hNEIL1 (K242) glycosylase suggests that the substrate recognition loop is disordered. Nevertheless, the partial electron density observed at the active-site for loop residue Lys242 reveals that the pre-cleavage intermediate resembles the residue ‘242-in conformation’ associated with active catalysis (58). The observation of Glu6-mediated H-bonding contacts involving the amide nitrogen of the Gly2–Glu3 peptide bond, and the Gly2 α-amine and urea lesion (Figure 4) provides insight concerning the formation of this pre-cleavage complex. Possibly, these additional H-bonding contacts prevent the correct orientation of the urea moiety in the active site, thus preventing subsequent steps in the proposed mechanism (Scheme 3).

The present pre-cleavage complex in the BER pathway (Scheme 3) differs from prior structures of glycosylases which either examined complexes stabilized by chemical reduction prior to crystallization (95,96) or revealed post-excision complexes. As an example of the latter, a post-excision complex of the monofunctional uracil DNA glycosylase UdgX revealed the conjugation of AP sites through residue H109, while the excised uracil remained in the active site (97). In that instance, base excision was postulated to occur via a S_N_1-like mechanism in which cleavage of the glycosidic bond occurred before nucleophilic attack at C1′ (97). That mechanism differs from the scenario observed in the present pre-excision urea-bound hNEIL1 complex (Scheme 3; Figures 3 and 4) and other substrate-bound structures of the hNEIL1/Fpg glycosylases (47,58,96). The structure of a product complex with MBD4, a helix-hairpin-helix (HhH) glycosylase, reported to be involved in active DNA demethylation (98), also revealed a post-excision intermediate, but before nucleophilic attack at C1′ (98). In that complex, C1′ would be anticipated to exist transiently as a carbocation or an oxocarbenium ion.

**Figure 6.**
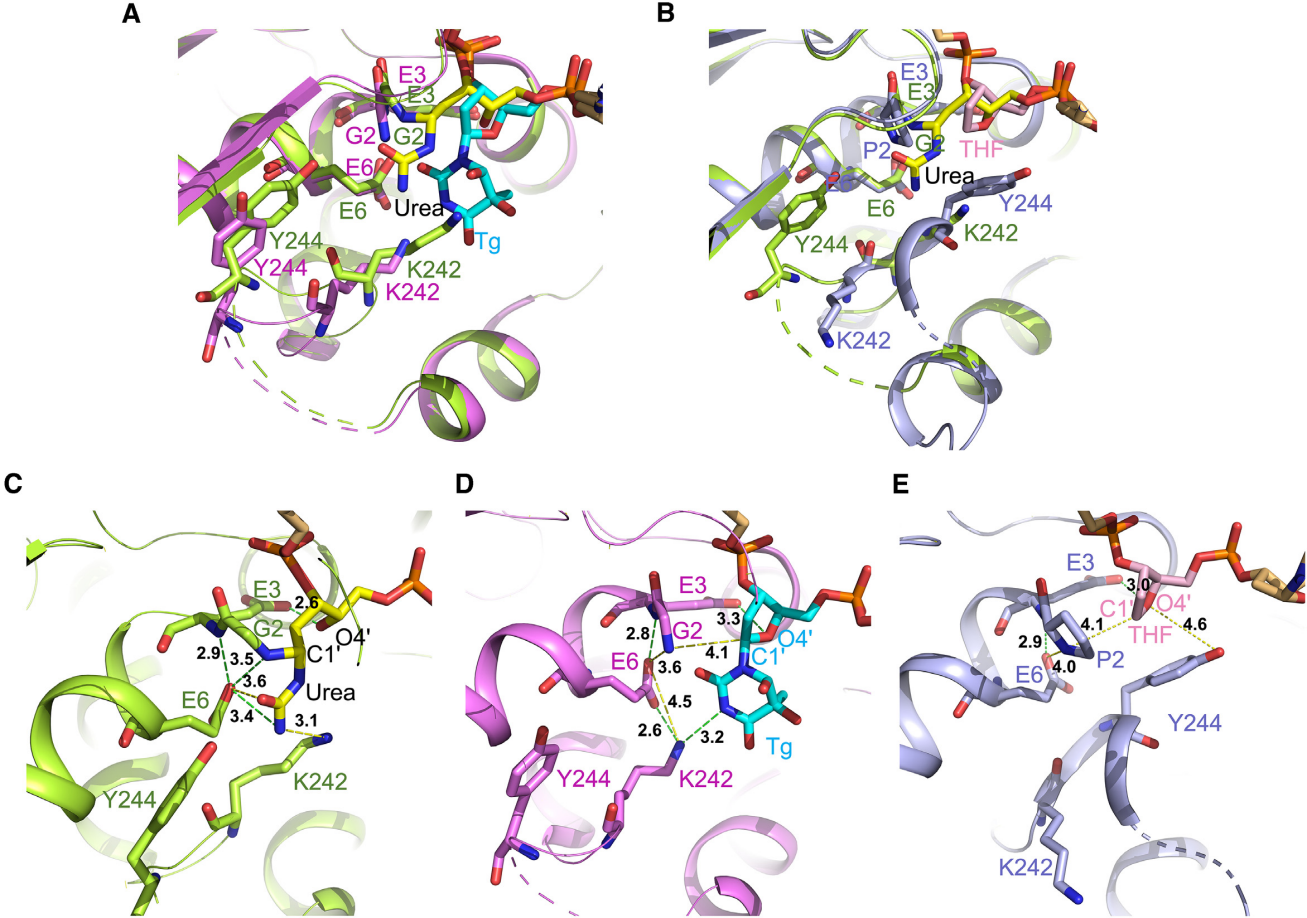
Comparison between the active sites in the crystal structures of CΔ100 P2G hNEIL1 (K242) bound to different ds DNAs. (**A**) Overlay of active sites in the complexes with urea- (green) and Tg- (magenta) containing DNA (present study and PDB id: 5ITX). In both, the ‘242-in conformation’ is observed. (**B**) Superimposed complex structures of the urea-(green) and THF-(blue) containing DNA (present study and PDB id: 5ITY). In the THF-bound complex structure, the ‘244-in conformation’ is highlighted. (**C**) View of the active site with distances for H-bonded interactions (green dotted lines) between flipped ring-opened deoxyribose-urea lesion (covalent intermediate) (yellow) and protein residues (green) atoms in P2G hNEIL1 (K242)-urea bound structure. The Gly2 α-amine nitrogen is covalently linked to the deoxyribose C1′. (**D**) View of the active site with distances for H-bonded interactions (green dotted lines) between flipped ring-closed deoxyribose-Tg lesion (cyan) and protein residue (magenta) atoms in the P2G hNEIL1 (K242)-Tg bound structure. For the Tg lesion, the Gly2 α-amine nitrogen is 4.1 Å from the deoxyribose C1′ atom. The distance between the Glu3 carboxylate and deoxyribose O4′ is greater for the Tg lesion than the urea lesion. (**E**) View of the active site with distances for H-bonded interactions (green dotted lines) between the flipped THF lesion (pink) and protein residues (blue) in the hNEIL1 (K242)-THF bound structure. The substrate recognition loop residue Tyr244 orients towards the THF moiety and caps the deoxyribose ring through its aromatic side chain. All H-bonded (green dotted lines) and non-bonded (yellow dotted lines) distances highlighted as numbers are in [Å].

Data reported by Zhu *et al.* (47) (Figure 6) showed that loop residue Tyr244, not Lys242, pointed towards the THF moiety, capping the deoxyribose through its aromatic side chain. This was the ‘residue 244-in conformation’, postulated to be catalytically inactive (58). While in that structure, the THF oxygen is closer to the Glu3 carboxylate as compared to the Tg-bound structure, it is farther away than in the present urea-bound structure (Figure 6). The inability of the CΔ100 P2G hNEIL1 (K242) glycosylase to incise Tg-containing DNA (Supplementary Figure S9 in the Supplementary Data) is consistent with its structure complexed with a Tg-containing DNA (47) (Figure 6). In that instance, the deoxyribose was in the furanose configuration and the N-terminal Gly2 amine was 4.1 Å from the deoxyribose C1′, while the Tg lesion remained intact.

### Residual activity of the CΔ100 P2G hNEIL1 (K242) glycosylase

The observation that CΔ100 P2G hNEIL1 (K242) glycosylase retains a residual activity against urea lesions and AP sites is consistent with reports that the P2G and P2T mutants of *E. coli* Fpg exhibit 10% of wt activity against Fapy-dG lesions (99). The observation that incision of urea-containing DNA using the CΔ100 P2G hNEIL1 (K242) occurred for ∼2 min and then plateaued (Figure 5) even when only ∼30% of the initial DNA substrate was excised (Figure 5) is intriguing. The present results confirm that urea lesions exist in DNA as a mixture of species, presumably α and β anomers, that interconvert slowly at pH 7.4 (Figure 1 and Supplementary Figure S4 in the Supplementary Data). A possible explanation is that the CΔ100 P2G hNEIL1 (K242) glycosylase exhibits stereoselective activity against urea lesions. In contrast, wt hNEIL1 completely excises urea lesions (Figure 2), so any stereospecific selectivity against urea lesions is likely a peculiar feature of the CΔ100 P2G hNEIL1 (K242) glycosylase. The potential for stereoselective excision of oxidative DNA damage by members of the Fpg/Nei family of glycosylases draws precedence from reports that wt hNEIL1 preferentially cleaves β anomers of alkylated Fapy-dG lesions (39,42). Other studies have reported stereoselective processing by hNEIL1 of Tg (26–28,30) and hydantoin Sp lesions (32). The basis for stereoselective rates of incision could include steric interference, altered hydrophobicity in the active site pocket or subtle changes in electrostatic forces.

## CONCLUSIONS

The urea lesion exists in DNA as a mixture of α and β anomers. It is an efficient substrate for both unedited (K242) and edited (R242) forms of the hNEIL1 glycosylase. Unlike Tg lesions, the excision efficiency of urea lesions is independent of editing of the glycosylase. The wt hNEIL1 likely excises urea lesions utilizing a mechanism like that for excision of Tg lesions (47,58). The structure of a pre-cleavage intermediate with the mutant CΔ100 P2G hNEIL1 (K242) glycosylase illustrates the role of active site residue Glu3 in protonation of deoxyribose O4′ and activation of the anomeric C1′ for nucleophilic attack. We surmise that H-bonded interactions involving Glu6, Gly2 and the urea lesion may prevent subsequent proton transfer steps necessary for base excision involving Lys242, resulting in the trapping of this pre-excision complex. Biochemical analyses confirm that the CΔ100 P2G hNEIL1 (K242) glycosylase retains residual activity for urea lesions. Overall, these findings provide additional insight into the mechanism and chemical biology of the wt hNEIL1 glycosylase.

## DATA AVAILABILITY

Atomic coordinates and structure factors for the reported crystal structures have been deposited with the Protein Data bank under accession number 8FTJ. Raw diffraction data have been deposited with SBGrid (DOI: 10.15785/SBGRID/909) under accession code 909.

## Supplementary Material

gkad164_Supplemental_File
