# Supplementary material for: Base excision repair of the N-(2-deoxy-d-erythro-pentofuranosyl)-urea lesion by the hNEIL1 glycosylase
Source: Nucleic Acids Res. 2023 Apr 4;51(8):3754–69. doi: 10.1093/nar/gkad164 (PMC10164570; doi:10.1093/nar/gkad164)
Supplement: gkad164_Supplemental_File [file gkad164_supplemental_file.docx]

**Base Excision Repair of the *N*-(2-Deoxy-D-*erythro*-pentofuranosyl)-urea Lesion by the hNEIL1 Glycosylase**

Rachana Tomar^1‡^, Irina G. Minko^2‡^, Pankaj Sharma^3^, Andrew H. Kellum, Jr.^1^, Li Lei^4^, Joel M. Harp^4^, Tina M. Iverson^3^, R. Stephen Lloyd^2,5^, Martin Egli^†,4^*, and Michael P. Stone^†,1,^*

**Supplementary Data**

^1^Department of Chemistry and the Vanderbilt-Ingram Cancer Center, Vanderbilt University, Station B Box 351822, Nashville, TN 37235

^2^Oregon Institute of Occupational Health Sciences, Oregon Health & Science University, 3181 SW Sam Jackson Park Rd., Portland, OR 97239

^3^Department of Pharmacology, Vanderbilt University, Nashville, TN 37232

^4^Department of Biochemistry, School of Medicine, and the Vanderbilt-Ingram Cancer Center, Vanderbilt University, Nashville, TN 37232

^5^Department of Molecular and Medical Genetics, Oregon Health & Science University, 3181 SW Sam Jackson Park Rd., Portland, OR 97239

*To whom correspondence should be addressed. Tel: M.E. +1 615-343-8070; M.P.S. +1 615-322-2589; Email: M.E. [martin.egli@vanderbilt.edu](mailto:martin.egli@vanderbilt.edu); M.P.S, [michael.p.stone@vanderbilt.edu](mailto:michael.p.stone@vanderbilt.edu); ORCID ID: M.E. <https://orcid.org/0000-0003-4145-356X>; M.P.S. <https://orcid.org/0000-0002-0922-0216>

^†^Joint Authors

^‡^Contributed Equally

**Figure S1.** Reversed phase-HPLC chromatogram of 5′-d(CGTCCAXGTCTAC)-3′ (X = urea lesion) (UV absorbance monitored at 254 nm) showing the presence of transiently separable chromatographic peaks (Peak 1 and Peak 2). Upon isolation, the peaks underwent re-equilibration to the mixture. These species were identified as α and β anomers of the 2′-deoxyribosyl ring for the urea lesion.

**
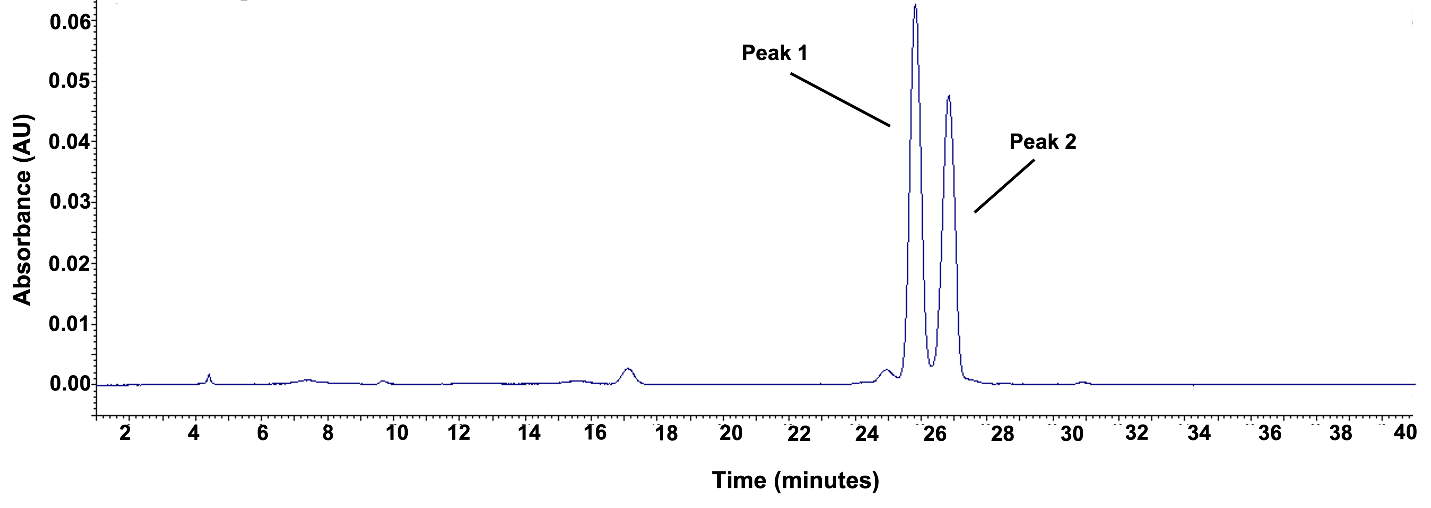
**

| **Fractions** | **Elution Time (min)** | **Area** | **Area %** |
| --- | --- | --- | --- |
| **Peak 1** | 25.8 | 1.54 x 10^7^ | 47.3 |
| **Peak 2** | 26.8 | 1.30 x 10^7^ | 39.9 |

**Figure S2.** Reversed phase-HPLC chromatogram of 5′-CXGA-3′ (X = urea lesion) (UV absorbance monitored at 254 nm) showing the presence of transiently separable chromatographic peaks. Upon isolation, the peaks underwent re-equilibration. These species were identified as α and β anomers of the 2′-deoxyribosyl ring for the urea lesion.


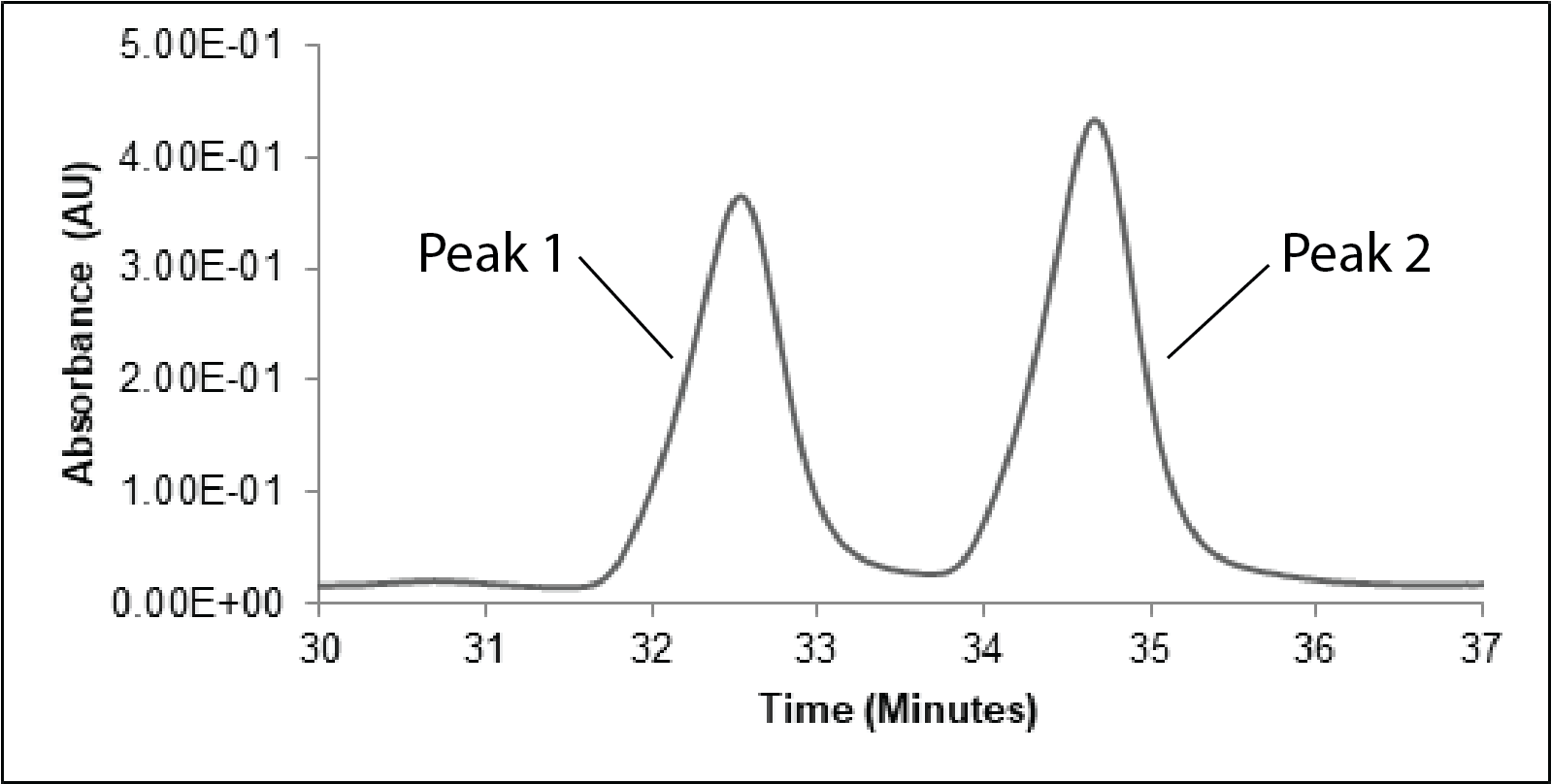


| **Fractions** | **Elution Time (min)** | **Area** | **Area %** |
| --- | --- | --- | --- |
| **Peak 1** | 32.6 | 1.48 x 10^7^ | 35.2 |
| **Peak 2** | 34.7 | 1.71 x 10^7^ | 41.0 |

**Figure S3.** FPLC chromatograms showing purification profiles monitored at 280 nm UV absorbance for (A) CΔ100 P2G hNEIL1 (K242) and (B) CΔ100 P2G hNEIL1 (K242) with ds DNA containing the urea lesion and (C) 4-12% SDS PAGE showing protein ladder (Lane 1), purified CΔ100 P2G hNEIL1 (K242) (Lanes 2 and 3) (in the absence of glycerol) and CΔ100 P2G hNEIL1 (K242) with ds DNA containing the urea lesion (Lanes 4 and 5). Purified fractions shown in the gel figure were collected from fractions bracketed by red lines in the respective chromatograms.


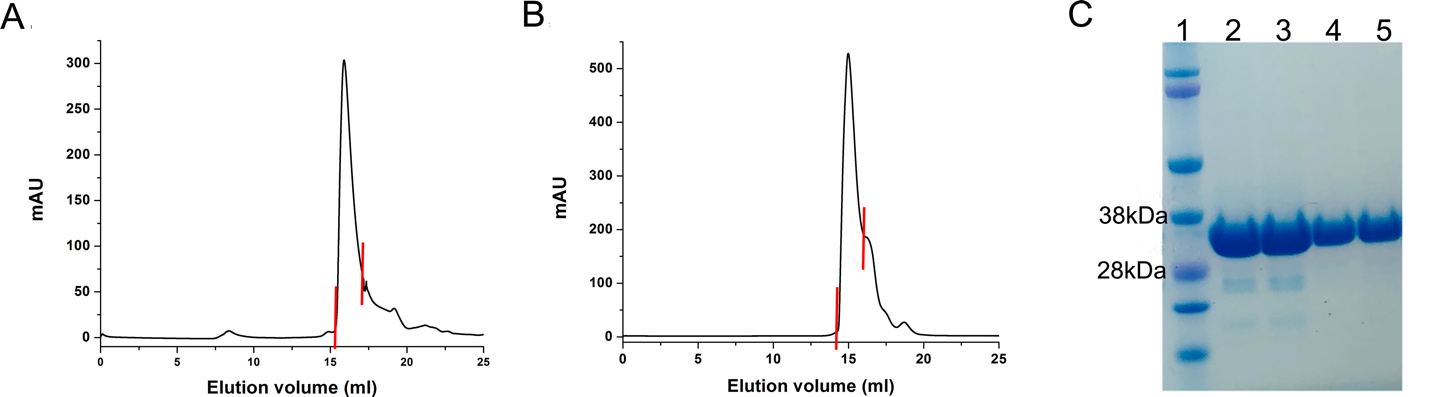


**Figure S4. A.** Chemical structure of the 2′-deoxyribosylurea and oligodeoxynucleotide sequence used in NMR studies to determine the configurational and conformational interconversions associated with the urea lesion. The NMR studies were conducted on a sample containing an anomeric mixture of the urea lesion. **B.** TOCSY spectrum showing the correlation between the NH and H1′ protons of X^2^ in 5′-C^1^X^2^G^3^A^4^-3′ where X= Urea lesion. **C.** ^1^H NMR spectrum showing the ^3^*J* coupling constants between the NH and H1' protons.


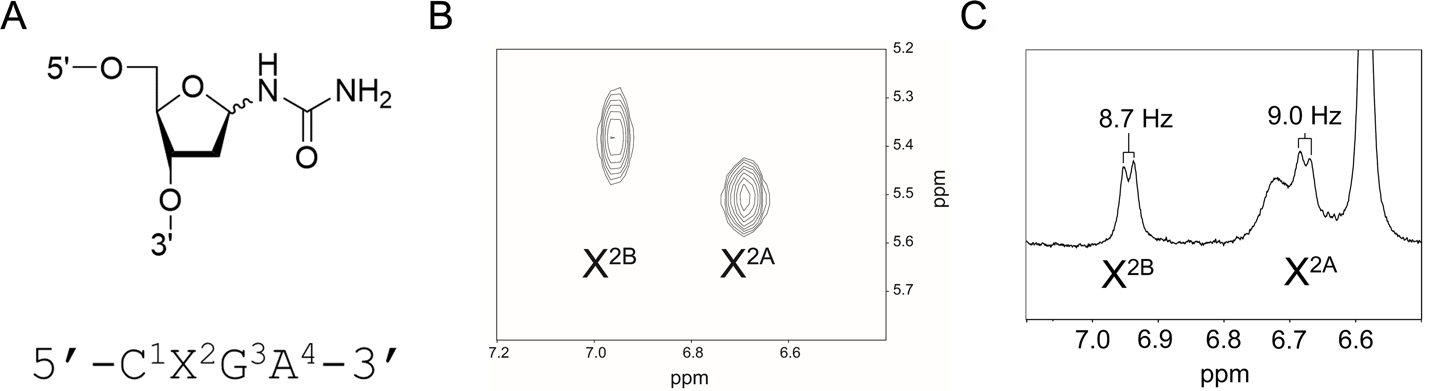


**Figure S5.** Comparison of the refined crystal structures of CΔ100 P2G hNEIL1 (K242) bound to the urea-containing ds DNA in *P*4_1_ and *P*4_1_22 space groups. The 2Fo-Fc electron density maps (contoured at 1σ) (dark blue mesh) of protein residues (green) Gly2, Glu3, Glu6, flipped nucleotide (yellow) at the active-site and loop residues, Gly240, Gly241, Lys242 and Tyr244 in *P*4_1_ space group and *P*4_1_22 space group are shown in **A and B**, respectively**.** Additionally, the Fo-Fc electron density maps (contoured at 3σ) (dark green mesh) to the position of truncated loop residues, Gly240, Gly241, Lys242 and Tyr244 (near active-site) in *P*4_1_ space group and in *P*4_1_22 space group are shown in **C and D**, respectively. **E.** Superimposed crystal structures of CΔ100 P2G hNEIL1 (K242) bound to the urea-containing ds DNA in *P*4_1_ and *P*4_1_22 space groups.

**
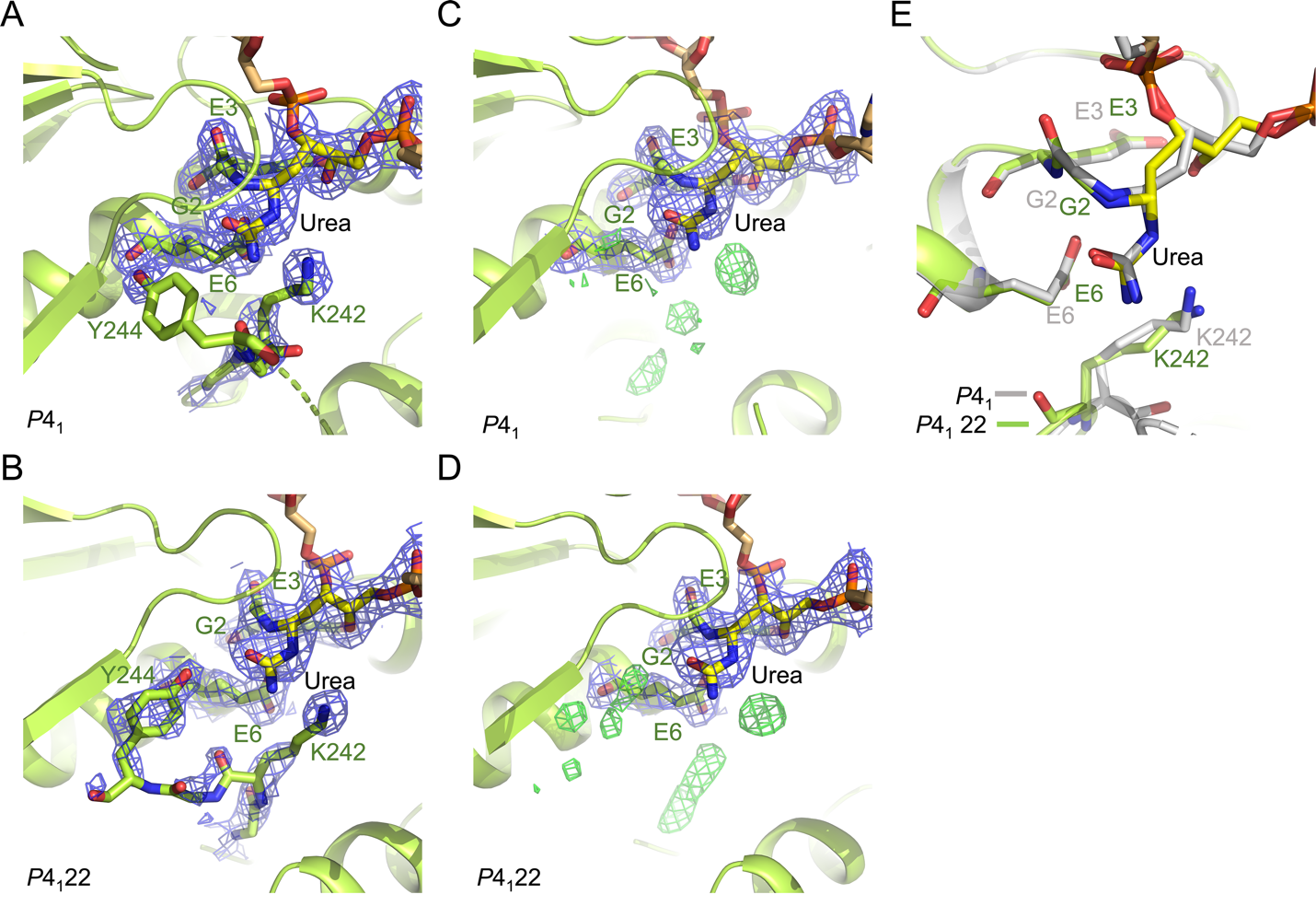
**

**Figure S6.** Refinement of model structures for the *R* (A) and *S* (B) diastereomers at the chiral C1′ of the urea lesion complexed with the CΔ100 P2G hNEIL1 (K242) glycosylase. The 2Fo-Fc Fourier sum and Fo-Fc difference electron density maps (contoured at 1σ (grey) and 3σ (green), respectively) of protein residues Gly2 and Glu3 and the flipped nucleotide at active site are shown. The *R* diastereomer fits the electron density better as indicated from Fo-Fc electron density maps in (A), suggesting that this pre-cleavage complex is derived from the β-anomer of the urea lesion. (C) Overlay of *R* and *S* diastereomers structures showing distances between the urea carbonyl oxygen and the Gly2 amine group.

**
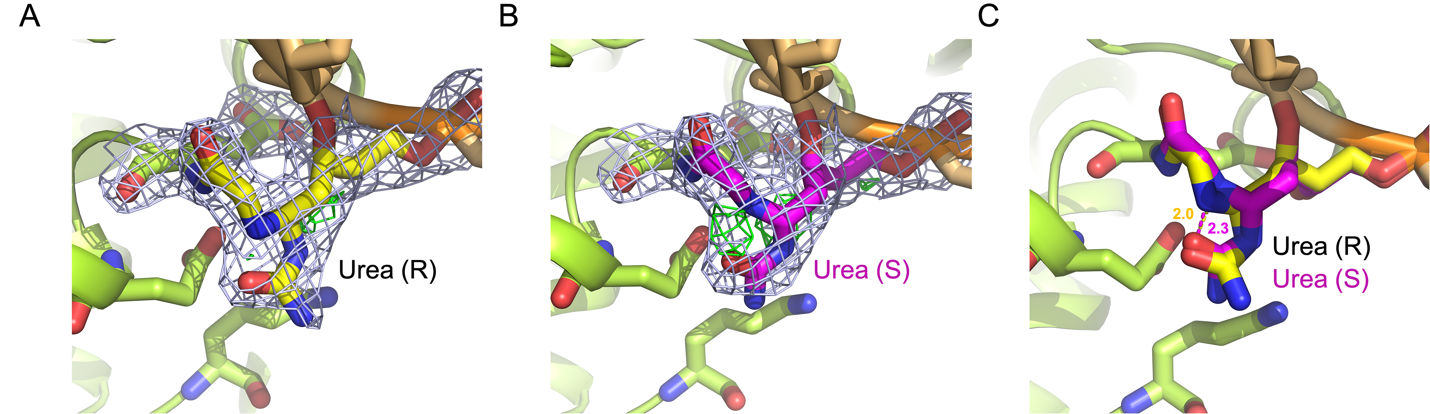
**

**Figure S7.** The 2Fo-Fc sum electron density maps (contoured at 1σ) (blue) showing modeled structures of (A) amino and (B) imino tautomers of the urea lesion in the active-site of the CΔ100 P2G hNEIL1 (K242) glycosylase. (C) Table of B-factor values for the urea atoms for the two tautomers.

**
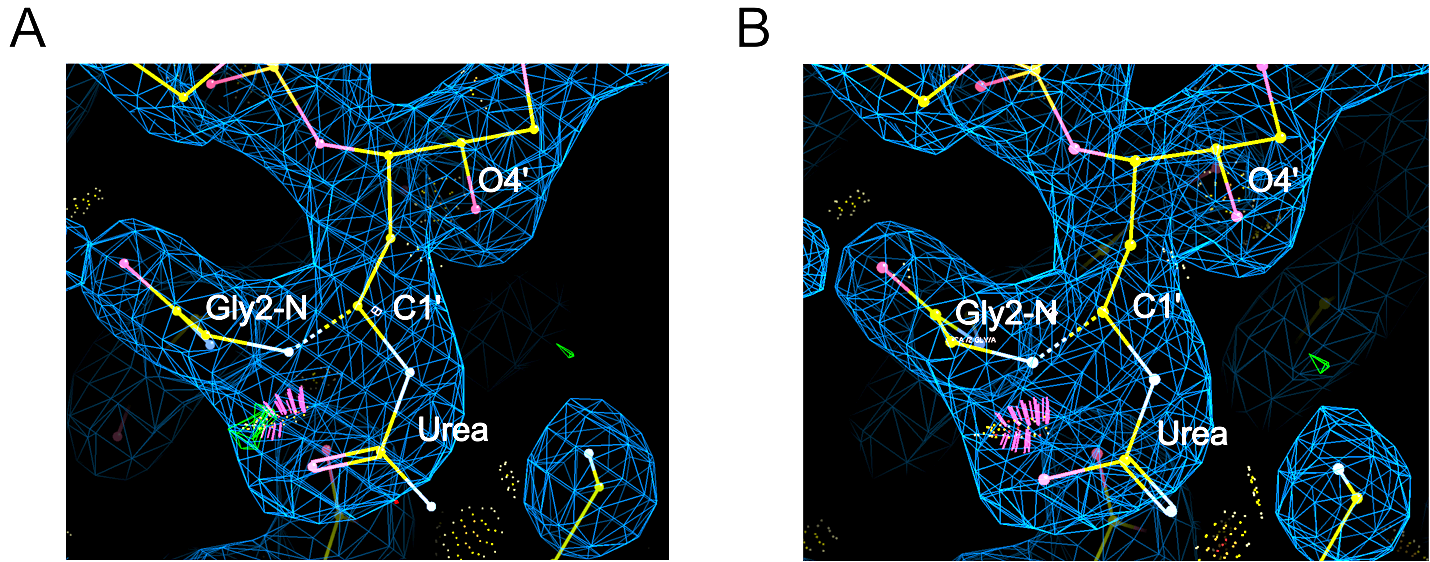
**

C

| B-factor [Å^2^] | | |
| --- | --- | --- |
| Urea atoms | amino tautomer | imino tautomer |
| =O or -OH | 32.4 | 30.3 |
| -NH_2_ or =NH | 57.2 | 53.7 |
| >NH | 29.5 | 27.3 |
| >C= | 39.6 | 36.8 |

**Figure S8.** Overview of protein-DNA interactions based on an analysis with PDBsum (1) and the DNA duplex. **A.** Interactions as seen in the crystal structure. **B.** Diagram showing interactions in an unwound ladder-like fashion. Residues are numbered according to the coordinates deposited in the PDB with accession code 8FTJ. H-bonds are shown with green dotted lines and other non-bonded contacts are shown with brown dotted lines (cut-off 3.35 Å). The urea lesion is depicted with orange sticks and water-mediated interactions are indicated with cyan spheres.

**Figure S8 A.**


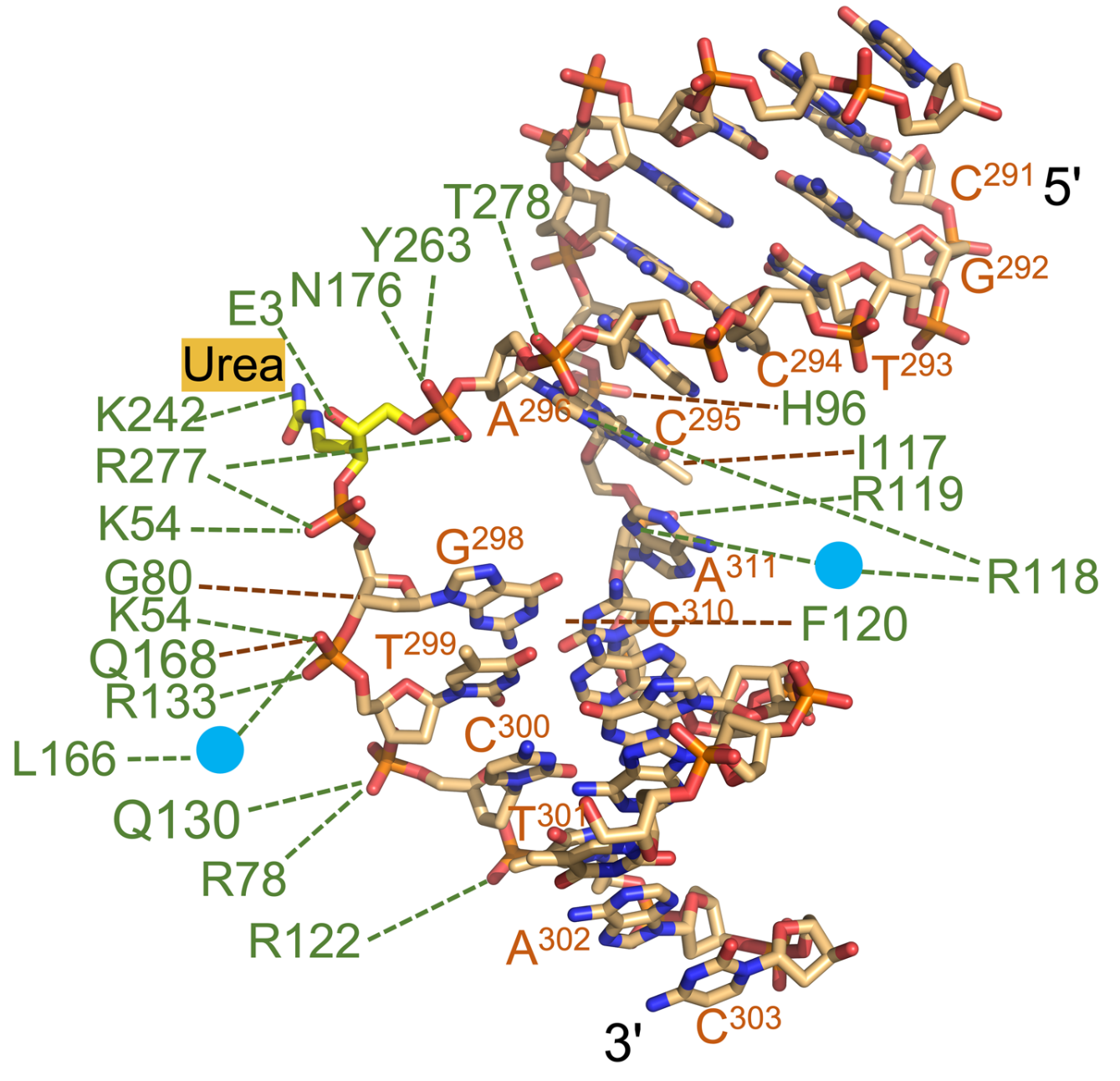


**Figure S8 B (See legend on previous page).**


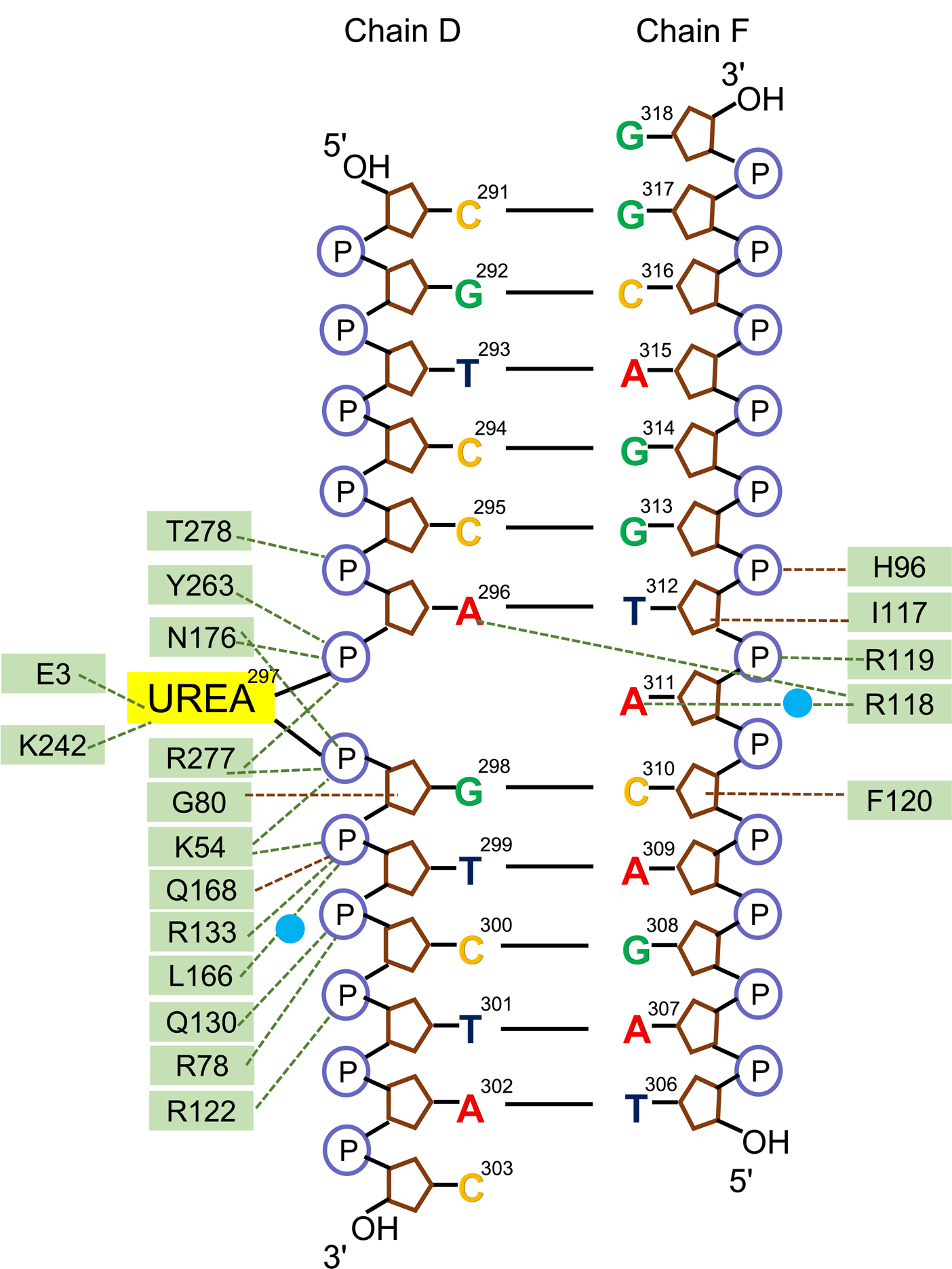


**Figure S9.** Activity assays for the CΔ100 P2G hNEIL1 (K242) glycosylase. Assays were conducted with TAMRA-conjugated DNA substrates as shown schematically in panel A. The ability of the CΔ100 P2G hNEIL1 (K242) glycosylase to incise DNA containing THF (B), Tg (C), or an AP site (D, E) was tested. Control assays contained APE1 (B), edited hNEIL1 (R242) (C, D, and F), or Fpg (G).

**
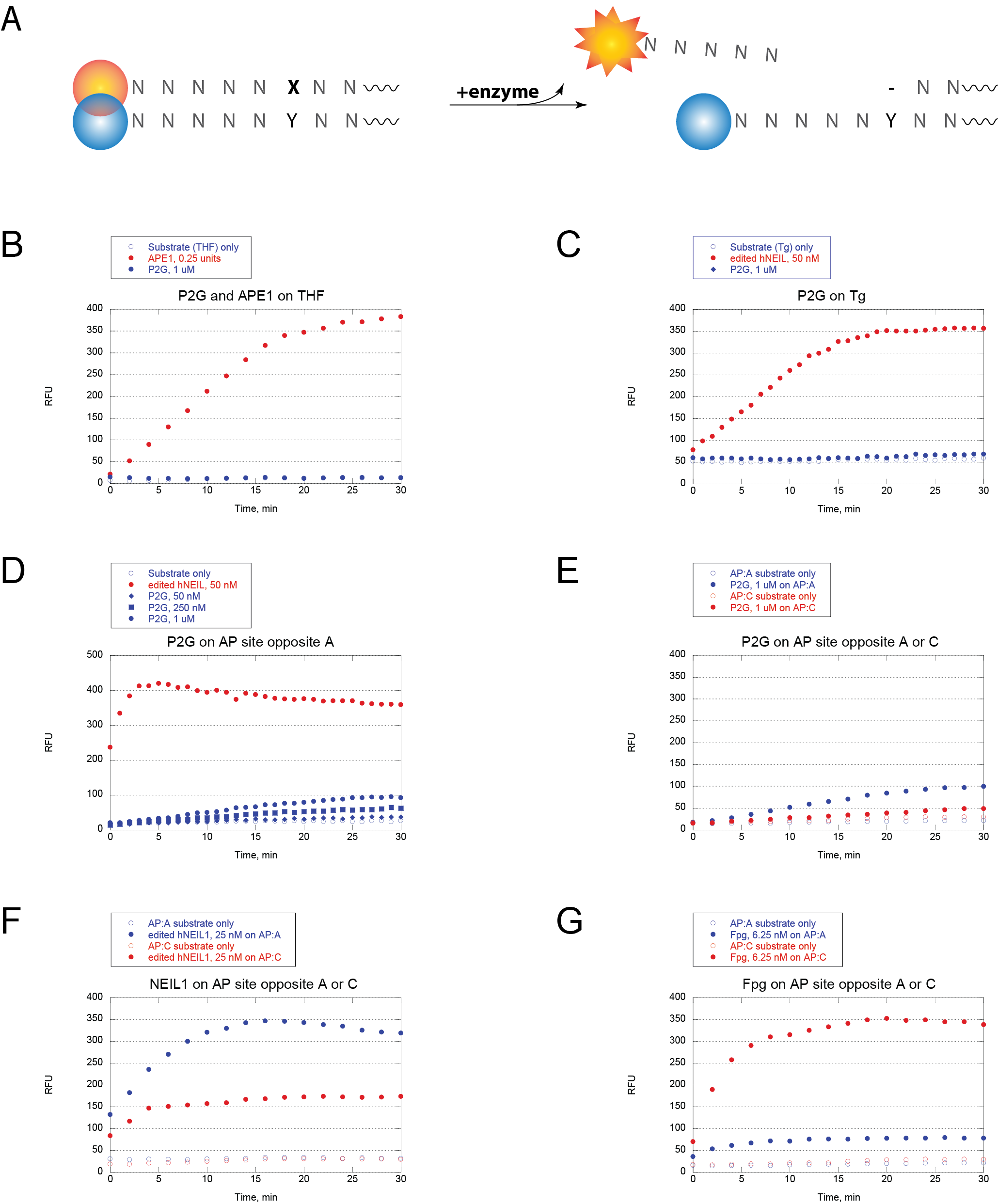
**

REFERENCES

1. Laskowski, R.A., Jablonska, J., Pravda, L., Varekova, R.S. and Thornton, J.M. (2018) PDBsum: Structural summaries of PDB entries. *Protein Sci*, **27**, 129-134.
